# STOML2 potentiates metastasis of hepatocellular carcinoma by promoting PINK1-mediated mitophagy and regulates sensitivity to lenvatinib

**DOI:** 10.1186/s13045-020-01029-3

**Published:** 2021-01-14

**Authors:** Yahui Zheng, Chong Huang, Lu Lu, Kangkang Yu, Jing Zhao, Mingquan Chen, Lu Liu, Qingfeng Sun, Zhifei Lin, Jianming Zheng, Jinhong Chen, Jubo Zhang

**Affiliations:** 1 Department of Infectious Diseases, Huashan Hospital, Fudan University, Shanghai, 200040 China; 2 Center of Liver Diseases, Huashan Hospital, Fudan University, Shanghai, 200040 China; 3 Department of General Surgery, Huashan Hospital, Fudan University, Shanghai, 200040 China; 4 Department of Infectious Diseases, Ruian People’s Hospital, Ruian, 325200 China

**Keywords:** Stomatin-like protein 2 (STOML2), Hepatocellular carcinoma (HCC), PTEN-induced putative kinase 1 (PINK1), Mitophagy, Lenvatinib

## Abstract

**Background:**

Dysregulation of both mitochondrial biogenesis and mitophagy is critical to sustain oncogenic signaling pathways. However, the mechanism of mitophagy in promoting hepatocellular carcinoma (HCC) progression remains poorly understood. In this study, we investigated the clinical significance and biological involvement of mitochondrial inner membrane protein STOML2 in HCC.

**Methods:**

STOML2 was identified by gene expression profiles of HCC tissues and was measured in tissue microarray and cell lines. Gain/loss-of-function experiment was applied to study the biological function of STOML2 in HCC. Flow cytometry, Western blotting, laser confocal microscopy, transmission electron microscopy, and co-immunoprecipitation were used to detect and analyze mitophagy. ChIP and luciferase reporter assay were conducted to evaluate the relationship between STOML2 and HIF-1α. The sensitivity to lenvatinib was assessed in HCC both in vitro and in vivo.

**Results:**

Increased expression of STOML2 was found in HCC compared with paired peritumoral tissues. It was more significant in HCC with metastasis and correlated with worse overall survival and higher probability of recurrence after hepatectomy. Upregulation of STOML2 accelerated HCC cells colony formation, migration and invasion. Mechanically, TCGA dataset-based analysis showed enrichment of autophagy-related pathways in STOML2 highly-expressed HCC. Next, STOML2 was demonstrated to interact and stabilize PINK1 under cellular stress, amplify PINK1-Parkin-mediated mitophagy and then promote HCC growth and metastasis. Most interestingly, HIF-1α was upregulated and transcriptionally increased STOML2 expression in HCC cells under the treatment of lenvatinib. Furthermore, higher sensitivity to lenvatinib was found in HCC cells when STOML2 was downregulated. Combination therapy with lenvatinib and mitophagy inhibitor hydroxychloroquine obtained best efficacy.

**Conclusions:**

Our findings suggested that STOML2 could amplify mitophagy through interacting and stabilizing PINK1, which promote HCC metastasis and modulate the response of HCC to lenvatinib. Combinations of pharmacologic inhibitors that concurrently block both angiogenesis and mitophagy may serve as an effective treatment for HCC.

## Background

Liver cancer was the sixth most common cancer type and the fourth leading cause of cancer-associated death, and hepatocellular carcinoma (HCC), usually develop from the hepatitis and hepatic cirrhosis, accounts for 75–85% of all primary cases [1]. Similar to other solid tumors, HCC has to overcome multiple stresses during progression, such as hypoxia, malnutrition, immunal cells cytotoxicity, and various treatments. In response to various stresses, mitochondria, the key organelle for energy production, reactive oxygen species (ROS) production and calcium buffering, will accumulate toxic metabolites [2, 3]. Selective elimination of dysfunctional mitochondria by mitophagy is an important process to maintain a functional network of tumor mitochondria, and breakdown products can be further used as bioenergetic intermediates to sustain unlimiting growth [4–6]. However, how mitophagy facilitates the turnover of damaged mitochondria for cell survival has not been fully elucidated.

Mitophagy is a specific form of selective autophagy, which aims to eliminate damaged mitochondria, prevent the accumulation of damaging mtDNA mutations and maintain the mitochondrial quality [4]. Recent insights into mitophagy suggest PTEN-induced putative kinase 1 (PINK1) and an E3 ubiquitin ligase Parkin play the central role in mitochondrial quality control [7]. PINK1 is a serine/threonine kinase, which can be imported into the mitochondrial inner membrane via outer/inner mitochondrial membrane translocase complex, and degraded by mitochondrial processing peptidase and mitochondrial inner protease presenilin associated rhomboid like (PARL) [8, 9]. When mitochondrial membrane potential is impaired by irradiation, ROS, or chemotherapeutic agents, PINK1 is stabilized on the outer mitochondrial membrane, leading to Parkin, Ub- and autophagy adaptor p62 recruitment to damaged mitochondria [10–13]. Certain mitochondrial proteins, including translocase of outer mitochondrial membrane complex (TOMM7) and PGAM5, have been demonstrated to retain and stabilize PINK1 in the mitochondrial outer membrane [9]. However, are there more mitochondrial relating proteins involved in PINK1 degradation and stabilization? The detailed mechanisms remain unclear.

Stomatin-like protein 2, also known as STOML2 or SLP2, identified as an inner mitochondrial membrane protein in human erythrocytes and many other tissues, shares a similar sequence with stomatin but lacks an NH _2_ -terminal hydrophobic domain, which distinguishes it from other family members [14, 15]. STOML2 is a regulator of mitochondrial biogenesis and ATP production [16]. There is a growing number of studies demonstrating that STOML2 is implicated in tumor progression and development. Using laser-capture tissue microdissection, two-cycle RNA amplification and genome-wide cDNA arrays, STOML2 was identified, in our previous study, as one of the significant differences among gene expression profiles of pure tumor cells of HCC with metastasis and metastasis-free HCCs as well as normal liver tissue [17]. So far the biological function and regulation mechanism of STOML2 in HCC was still poorly understood. Similar to prohibitin 2, an inner membrane mitophagy receptor, STOML2, is a member of superfamily of putative scaffolding proteins [18, 19]. Whether and how STOML2 involves in the mitophagy is still unknown.

Nowadays, tumor is regarded as a kind of chronic disease. HCC is usually derived from chronic liver injuries with extensive cytokines and uncontrolled angiogenesis, which not only complicates treatment choice, but also competes the effect of tumor progression on patient survival. Targeting angiogenesis has been introduced as a logical approach, and enormous innovative anti-angiogenic agents have been developed successfully [20]. However, facing the long-term therapeutic stress, the genetic instability brings in advantages in favor of tumor survival and drug resistance, such as high expression of molecules promoting mitophagy [10]. So far autophagy inhibitor, such as hydroxychloroquine, is under clinical evaluation (clinical trials NCT 03037437, NCT02013778). Hydroxychloroquine alone has shown limited effects, but the combination therapy is promising. Antiangiogenesis inhibitors, the drugs of first line treatment in advanced HCC, inhibit angiogenesis and result in severe hypoxia. Whether the reactive mitophagy is inevitable and the influences of mitophagy on acquired insensitivity to antiangiogenesis offer a fertile for in-depth study.

In this study, we found that, compared with peri-tumor tissues, STOLM2 was highly expressed in HCC and predicted a poor clinical prognosis. Both gain- and loss-of-function in vitro and in vivo assays indicated that STOML2 promoted HCC growth and metastasis. The pro-metastatic activity of STOML2 is most likely attributed to its interacting with and stabilizing PINK1, which further activate Parkin-mediated mitophagy in HCC cells. Notably, we demonstrated that lenvatinib upregulated the expression of STOML2 with HIF-1α dependent. Blockage of mitophagy in STOML2-highly expressed HCC enhanced the anti-HCC activity of lenvatinib both in vitro and in vivo, that provided a novel strategy to improve the clinical therapeutic efficacy of lenvatinib in HCC patients.

## Methods

### Clinical samples

Two sets of HCC samples were used in our study. The first set containing 48 HCC samples was used to analyze STOML2 expression at mRNA and protein level. The second set containing 227 HCC samples was used to analyze STOML2 protein expression and evaluate the correlation with clinicopathological features. The details are described in Additional file 1: supplementary materials and methods.

### Immunohistochemical (IHC) staining

IHC staining was performed using EnVisiontm system as previously described [21]. Antibodies applied in this experiment are listed in Additional file 2: Table S1. The detail procedures were presented in Additional file 1: supplementary materials and methods.

### Establishment of overexpression or knock-down cell lines

All transfections were performed using Lipofectamine™ 3000 (Invitrogen, L3000015) according to the manufacturer’s instructions. The respective primers sequence for STOML2 knock-down are shown in Additional file 2: Table S2. The details are described in Additional file 1: supplementary materials and methods.

### Immunofluorescence (IF) staining

All the HCC cells used were seeded on cover slides in 24-well plates, incubated overnight and then fixed in 4% paraformaldehyde for 15 min, permeabilized with 1% Triton X-100 for 5 min, blocked in 1% bovine serum albumin (BSA) for 60 min, and incubated with primary antibodies for 60 min at RT, followed by secondary antibodies for 60 min at RT. Nuclei were stained with 4′,6-diamidino-2-phenylindole dihydrochloride (DAPI, Cell Signaling Technology, #4083) at RT for 10 min. Photographs were captured with a laser confocal microscopy (Leica Microsystems AG). Antibodies applied in this experiment are listed in Additional file 2: Table S1.

### Immunoprecipitation and mass spectrometry (IP/MS)

SMMC-7721 cells transfected with STOML2-Flag were lysed in RIPA buffer and then loaded to Flag antibody and protein A/G agarose beads (Santa Cruz, sc-2003), the beads were washed with RIPA buffer for 5 times. Proteins complex was eluted using loading buffer, separated on SDS-PAGE gel and silver stained. Lysates from SMMC-7721 cells transfected with pENTER were used as control. Bands specific to the STOML2-Flag transfection were excised and subjected to mass spectrometry analysis on ABI 4700 MALDI TOF**.**

### Ubiquitination assay

Three days after infection with lentiviruses containing control, STOML2, or STOML2-specific shRNAs, proteosomal degradation was blocked by treating the cells with 20 μM MG132 for 6 h. Cells were lysed with 150 μL of denaturing lysis buffer (50 mM Tris–Cl at pH 6.8, 1.5% SDS), and then collected by scraping followed by boiling for 15 min. Ninety microliters of the denatured protein samples was added to 1 mL of EBC/BSA buffer (50 mM Tris–Cl at pH 6.8, 180 mM NaCl, 0.5% NP40, 0.5% BSA) and incubated with anti-PINK1 antibody or anti-FLAG antibody overnight and with protein A/G beads for 1 h at 4 °C. Ubiquitin antibody was used to detect poly-ubiquitinated PINK1 in the IP samples.

### Tumor xenografts in nude mice

All experimental procedures involving animals were approved by The Animal Care and Use Committee of Fudan University, China. Five-week-old nude mice (BALB/c) were randomly divided into indicated groups (*n* = 5 per group) before inoculation or injection. HCCLM3-shNC and HCCLM3-shSTOML2 cells were subcutaneously injected into the mice (1.0 × 10^7^ cells/mouse) to form the subcutaneous model. For the xenograft model, subcutaneous tumors were removed and dissected into 1 mm^3^ sections, which were incubated into the liver parenchyma of nude mice. For drug treated groups, mice were injected intraperitoneally with hydroxychloroquine (HCQ, 50 mg/kg), lenvatinib (LV, 5 mg/kg or 10 mg/kg), combination with lenvatinib (5 mg/kg) and HCQ (50 mg/kg) or saline as control. The mice were sacrificed at a time-defined endpoint and tumor volume, weight, and number of metastatic nodules were assessed by double-blinded evaluation. The details are presented in Additional file 1: supplementary materials and methods.

### Statistical analysis

Statistical analysis was performed with SPSS 22.0. All the data were expressed as mean ± standard deviation (SD). Kaplan–Meier analysis was used for survival analysis, and the log-rank test was chosen to compare the difference. Cox proportional hazards regression analyses were adopted for multivariate analysis. Pearson test or Fisher’s exact test were employed to compare qualitative variables, while Student *t* test or One-way ANOVA were used for quantitative variables. *P* < 0.05 was considered statistically significant.

For more details and other methods, see Additional file 1: supplementary materials and methods.

## Results

### STOML2 is upregulated in HCC tissues and correlates with poor prognosis

**Fig. 1:**
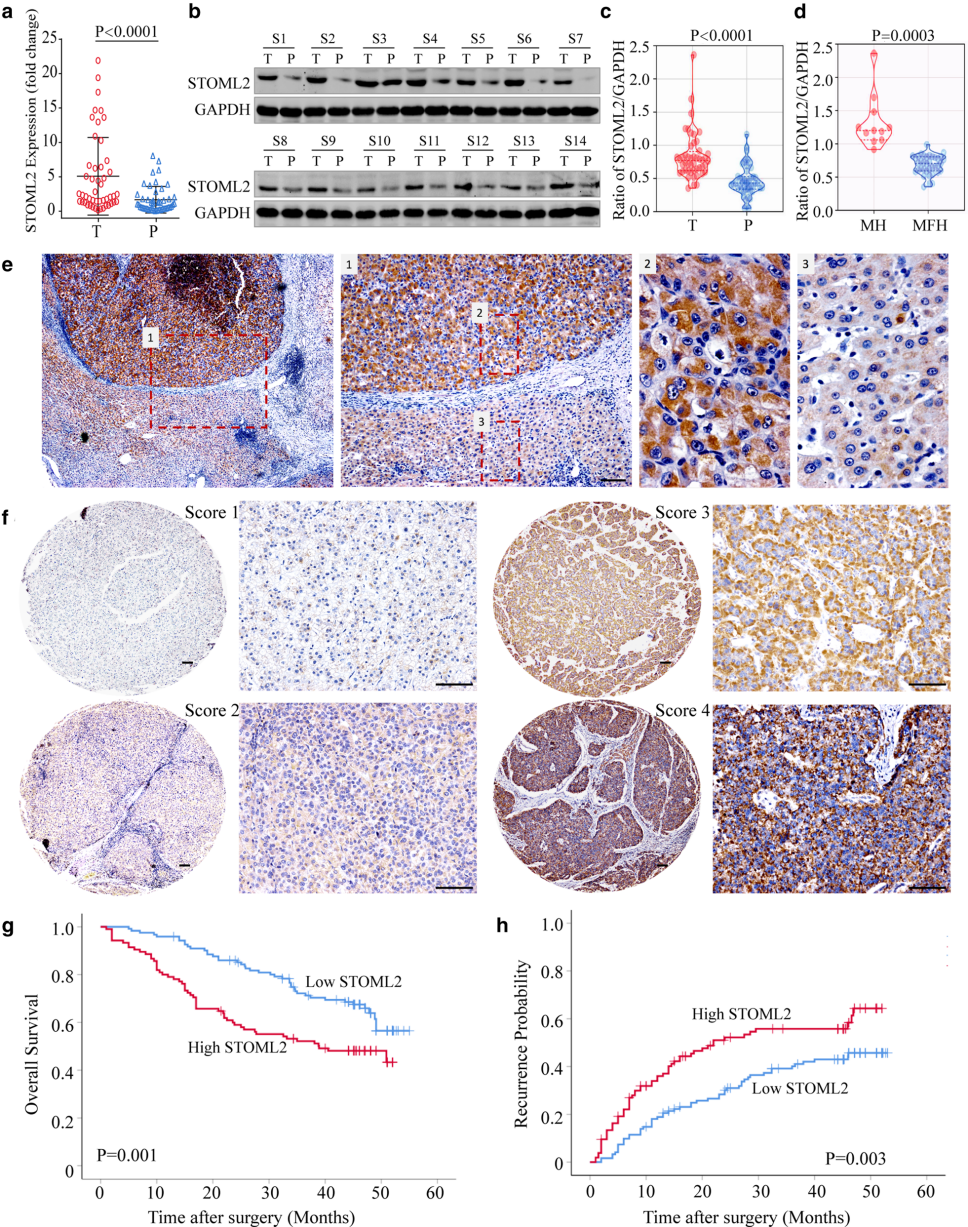
STOML2 expression is upregulated in HCC tissues and predicts a poor prognosis. **a-e** The mRNA and protein level of STOML2 in 48 matched HCC tissues and adjacent non-tumor liver tissues was detected by qRT-PCR (**a**), Western blot (**b**), and immunohistochemical staining assay (**e**), representative bands of the STOML2 in HCC tissues and peri-tumor liver tissues were shown. The expression of STOML2 was normalized against GAPDH, according to the intensity of each lane with the use of computerized image system (Image-Pro Plus 6.0), and it was much higher in HCC tissues in contrast to the matched adjacent non-tumor liver tissues (**c**). Compared with metastasis-free HCC, the expression of STOML2 in metastasis HCCs was much higher (**d**). Representative immunohistochemical staining of STOML2 in HCC tissues and peri-tumor liver tissues were shown (Scale bar: 100 μm): 1, the view of both HCC and peri-tumor tissues; 2, the view of HCC tissue; 3, the view of peri-tumor tissue (**e**). **f** Scores indicate STOML2 levels in representative tumor tissues with immunohistochemical staining, which were calculated by intensity and percentage of stained cells (Scale bar: 100 μm). **g**, **h** Patients with high STOML2 expression have poorer OS (**g**) and higher probability of recurrence (**h**) compared with patients with low STOML2 expression: *T* tumor, *P* peri-tumor, *S* specimen, *MH* metastasis HCCs, and *MFH* metastasis-free HCCs.

To further demonstrate what we found in previous gene expression profiles analysis that STOML2 was highly expressed in HCC, especially with metastasis [17], quantitative real-time PCR (qRT-PCR), immunoblotting assay, and immunohistochemistry in 48 HCC tissues and corresponding peri-tumor liver tissues were carried out. The data showed that the expression of STOML2 in HCC was much higher than the adjacent non-tumor liver tissues on both mRNA and protein levels (Fig. 1a–e), which is identical to the statistical results of evaluated analyzing the mRNA expression level of STOML2 by the GEPIA database (Additional file 3: Figure S1A) [22]. Notably, in comparison with non-metastatic HCC tissues (*n* = 37), the expression of STOML2 was significantly higher (*P* = 0.0003) in metastatic HCC tissues (*n* = 11), which showed intrahepatic spreading or tumor invasion into blood vessel or bile ducts (Fig. 1d). These data showed a close association of STOML2 upregulation with HCC.

Then, we evaluated the above association of STOML2 and HCC as well as the prognostic value of STOML2 in HCC. Survival analysis based on the RNA sequencing expression data of the HCC patients (*n* = 369) from the TCGA database was performed first and the results indicated that patients with higher STOML2 expression had remarkably worse overall survival (OS, *P* = 0.011) and disease-free survival (DFS, *P* = 0.029) (Additional file 3: Figure S1B-C) [23]. Second, the immunohistochemistry study of STOML2 in consecutive tissue microarray with 227 HCC tissues was performed. These patients, among which 186 cases (81.9%) showed liver cirrhosis with hepatitis B background, were divided into high or low STOML2 expression groups according to the immunostaining scores (Fig. 1f). Patients with microvascular invasion and advanced TNM stage appeared to possess high STOML2 levels in primary HCC tissues. More importantly, when compared with low STOML2 expression group, the significantly worse OS (median OS: 38.9 months versus more than 55 months, log-rank = 10.3, *P* = 0.001) (Fig. 1g) and shortened time to tumor recurrence (TTR) (median TTR: 22 months versus 53 months, log-rank = 8.7, *P* = 0.003) (Fig. 1h) were found in STOML2 high expression group. Hepatitis B e antigen positive, high AFP level, large tumor size, microvascular invasion, multiple tumors, and advanced TNM stage were also found associated with worse OS and shortened TTR in univariate analysis (Table 1, Additional file 2: Table S3). To assess the correlation between high STOML2 level and other risk factors, a Cox proportional hazards analysis was performed. The results showed that high STOML2 level is an independent risk factor for worse OS (hazard ratio = 1.596, *P* = 0.026) and shortened TTR (hazard ratio = 1. 638, *P* = 0.011). Taken together, these data imply that upregulated STOML2 has a significant correlation with poor prognosis of HCC and it may contribute to HCC progression.

**Table 1:** Association between clinicopathological features, STOML2 expression and survival

Factors	OS				TTR			
	Univariate *P* value	Multivariate			Univariate *P* value	Multivariate		
		HR	95% CI	*P* value		HR	95% CI	*P* value
Hepatitis B e antigen: positive versus negative	0.038	1.547	1.033–2.318	0.034	0.013	1.709	1.169–2.498	0.006
AFP (≤ 400 versus > 400 ng/ml)	0.024			NS	0.005			NS
Tumor differentiation: low versus high	0.027	1.920	1.192–3.095	0.007	0.235			NA
Vascular invasión(yes vs*.* no)	< 0.001			NS	0.001			NS
Tumor size (≤ 5 vs. > 5 cm)	< 0.001	2.963	1.864–4.710	< 0.001	< 0.001	2.108	1.392–3.193	< 0.001
Tumor number (single versus*.* multiple)	0.004			NS	0.008			NS
UICC TNM stage (I vs. II vs*.* IIIA)	< 0.001	1.703	1.274–2.275	< 0.001	< 0.001	1.548	1.186–2.022	0.001
STOML2 (low vs. high)	0.001	1.596	1.057–2.410	0.026	0.003	1.638	1.120–2.395	0.011

*OS* overall survival, *TTR* time to recurrence, *CI*, confidence interval, *HR* hazard ratio, *NA* not adopted, *NS* not significant, *AFP* α-fetoprotein, *UICC* International Union Against Cancer, *TNM* tumor-node-metastasis, *STOML2* Stomatin-like protein 2

### STOML2 promotes proliferation, migration, invasion, and inhibits apoptosis of HCC cells

**Fig. 2:**
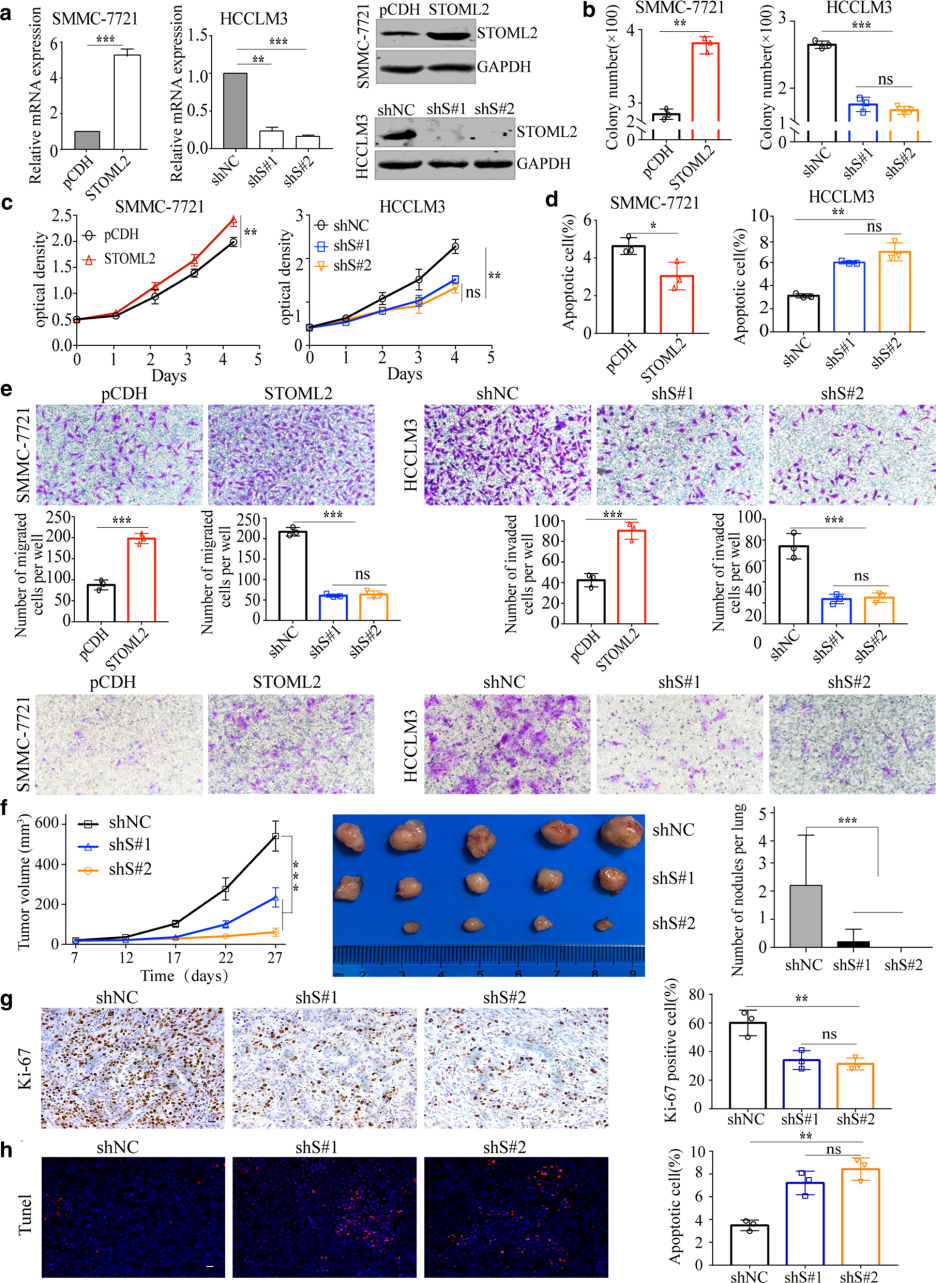
STOML2 promotes HCC cell proliferation, migration, and invasion and inhibits HCC cell apoptosis. **a** Overexpression of STOML2 in SMMC-7721 and knockdown in HCCLM3 were detected by qRT-PCR (left and middle panel) and Western blot (right panel). **b–e** The effects of STOML2 gain- or loss-of-function on in vitro proliferation (**b**, **c**), apoptosis (**d**), migration, and invasion (**e**) of SMMC-7721 and HCCLM3 cells were measured by colony formation assay, CCK8, flow cytometric analysis of Annexin-V/PI staining and transwell assays. **f** The dynamic change of tumor volume between HCCLM3-shNC and HCCLM3-shS#1, HCCLM3-shS#2 in subcutaneous models, were shown (left and middle panel). Downregulation of STOML2 significantly suppressed lung metastasis in tail vein injection model (right panel). **g** Representative immunohistochemical staining of Ki-67 in subcutaneous tumor tissues of mice was shown (Scale bar: 100 μm), and the number of cells positively stained with Ki-67 was calculated from three independent fields for each image. **h** Representative TUNEL staining in subcutaneous tumor tissues of mice was shown (Scale bar: 100 μm), and the number of cells positively stained was calculated from three independent fields for each image. *P < 0.05; **P < 0.01; ***P < 0.001; *ns*, no significance

Concerning the higher expression of STOML2 in HCC with metastasis, we evaluated the functions of STOML2 in HCC cells using both in vitro and in vivo assays. First, we examined the expression of STOML2 in HCC cell lines with different metastasis potential (Additional file 4: Figure S2A-B). The results of qRT-PCR and Western blot showed that high metastatic potential HCCLM3 and MHCC-97H cells expressed high levels of STOML2 versus low expression of STOML2 in low malignant potential SMMC-7721, Bel-7402 cells. Modulating the expression of STOML2 in HCC cells with lentivirus-mediated specific short hairpin (sh) RNAs or STOML^Flag^ (Fig. 2a), we found that over-expression of STOML2 in SMMC-7721 cells resulted in significant promotion of colony formation (Fig. 2b) and cell proliferation (Fig. 2c). Accordingly, the percentage of total (both early and late) apoptotic cells were obviously decreased (Fig. 2d, Additional file 4: Figure S2D).

To specifically address the important role of STOML2 in the migration and invasion of HCC cells, transwell assays were performed. The results suggested that the promoting migration and invasion role of STOML2 (*P* < 0.001) is much more significant than that on the proliferation (Fig. 2e). We then verified the influence of STOML2 on malignant potential in HCCLM3 with STOML2 specific shRNA#1 and shRNA#2 (shS#1, shS#2). Identical to upregulation of STOML2, STOML2-knockdown (KD) decreased the proliferation and colony formation, especially decreased migration and invasion significantly (Fig. 2c-e, Additional file 4: Figure S2C).

To confirm in vitro findings, in vivo experiments were carried out. In subcutaneous implantation nude mice models, tumor growth was monitored every 5 days. 20 days later, the nude mice injected with HCCLM3 cells transfected with negative control shRNA (shNC) were found to have much bigger tumor sizes than those injected with HCCLM3-shS#1 and HCCLM3-shS#2 cells (*P* < 0.001, Fig. 2f, left and middle panel). Moreover, we observed a significant reduction of Ki-67 expression by immunohistochemistry analysis and induction of apoptosis confirmed by TUNEL staining when STOML2 was knockdown (Fig. 2g–h). In tail vein injection lung metastasis models, one mouse in shS#1 group was found lung metastasis and none in shS#2 group, whereas the mean number of lung metastasis in shNC group was 2.2 per lung (Fig. 2f, right panel). In brief, these in vitro and in vivo gain- and loss-of-functional studies suggested the key role of STOML2 in promoting HCC growth and metastasis.

### STOML2 induces mitophagy in HCC cells under stresses

**Fig. 3:**
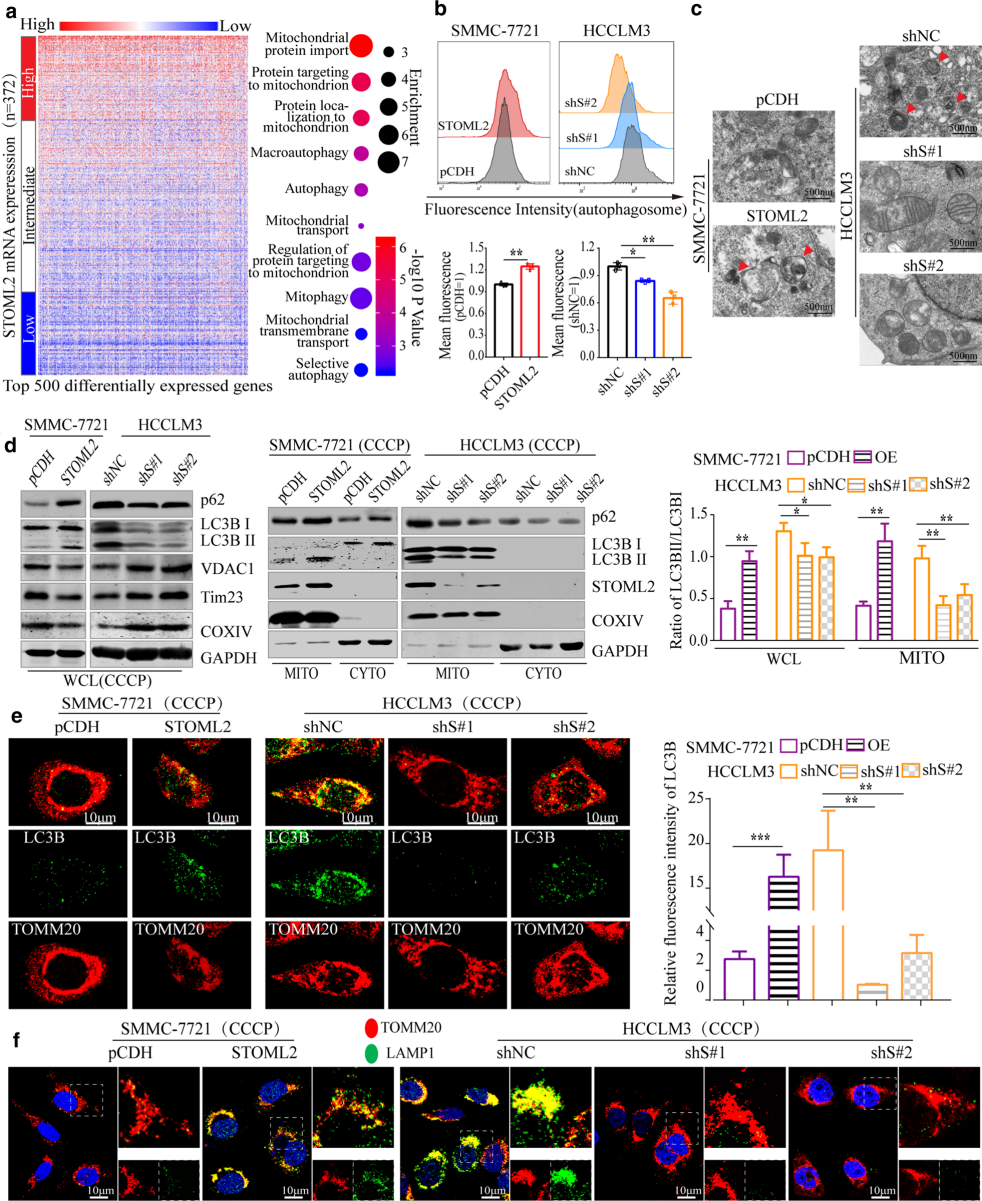
STOML2 promotes mitophagy in HCC cells under stress. **a** Expression of top 500 differentially expressed genes upregulated in HCC patients with different STOML2 expression and gene ontology term enrichment analysis for different biological processes controlled by differentially expressed genes among patients with high STOML2 expression. **b** The autophagic vacuoles were labeled with MDC and then detected by flow cytometry in SMMC-7721 and HCCLM3 cells. The fluorescence intensity of cells represented the autophagic level. **c** Representative TEM images depicted ultrastructure in SMMC-7721-pCDH or -STOML2-Flag and HCCLM3-shNC- or -shSTOML2. Red arrows indicated autophagic vacuoles. (Scale bars: 500 nm) **d** Under the treatment of CCCP (10 μM), total protein levels of p62, LC3B I/II and mitochondrial protein VDAC1, Tim23, COXIV were analyzed by Western blot. GAPDH was used as a loading control (left panel). Protein levels of STOML2, p62, and LC3B I/II in mitochondria were examined by purifying mitochondria from HCC cells. COX IV was used as a loading control for mitochondria (middle panel). Quantifying the ratio of LC3B II/LC3B I with image system in total and mitochondrial protein levels (right panel). **e**, **f** Confocal microscopy was performed to detect spatial co-localization of mitochondrial protein TOMM20 (red) with LC3B (green) (**e**, left and middle panel) and LAMP1 (green) (**f**) in SMMC-7721 and HCCLM3 control and derived cells under the treatment of CCCP (10 μM) for 4 h. (Scale bars:10 μm) The relative fluorescence intensity of LC3B are shown (**e**, right panel). *P < 0.05; **P < 0.01; ***P < 0.001; *ns* no significance, *WCL* whole cell lysate, *MITO* mitochondria

STOML2 has been identified as a mitochondrial inner membrane protein, belonging to a superfamily of putative scaffolding proteins, including Prohibitin 2, an inner membrane mitophagy receptor [19]. Thus, we speculated that STOML2 might be involved in autophagy in HCC, especially in mitophagy. To validate this hypothesis, we first assessed the transcriptomes of TCGA HCC patients with varying STOML2 expression and analyzed the top 500 differentially expressed genes in STOML2 high-expression versus STOML2 low-expression patients. The results showed that these genes were significantly enriched in biological processes including regulation of protein targeting to mitochondrial and autophagy (Fig. 3a). Next, monodansylcadaverine (MDC)-labeled autophagic vacuoles were detected by flow cytometry in vitro. The fluorescence intensity of SMMC-7721 with upregulation of STOML2 was significantly stronger than control cells while it dropped drastically in HCCLM3 with STOML2-KD (Fig. 3b). More bilayer membrane-bound autophagosomes were also observed by transmission electron microscopy (TEM) in those HCC cells with high STOML2 expression, which further supported the strong correlation between STOML2 and autophagy (Fig. 3c). The analysis of Western blot ulteriorly demonstrated that, in HCC cells with high STOML2 expression, autophagy marker cytosolic microtubule-associated protein 1A/1B-light chain 3 B (LC3B I, an important subtype of LC3 I) became conjugated phosphatidylethanolamine to form LC3B II. Consistently, STOML2-KD in HCC cells resulted in weakened LC3B lipidation under the treatment of carbonyl cyanide mchlorophenylhydrazone (CCCP) (Fig. 3d, left panel). More diffuse fluorescence of LC3 B were detected using immunofluorescence in HCC cells with high STOML2 expression (Fig. 3e).

An increase of mitochondrial protein TOMM20, COX IV, Tim23, and VDAC1 in HCC cells with STOML2-KD was demonstrated by Western blot or confocal laser microscope, indicating the accumulation of mitochondrial proteins. Furthermore, we purified mitochondria from HCC cells and examined the protein expression of p62 and LC3B II in mitochondria and cytoprotein, respectively. Much different to the weak change of them in cytoprotein, p62 and LC3B II increased significantly in mitochondria of SMMC-7721 with STOML2-overexpresion while decreased in HCCLM3 with STOML2-KD, respectively (Fig. 3d, middle and right panels). With confocal co-localization analysis, we found a substantial amount of TOMM20 predominantly co-localized with lysosome degradation marker LAMP1 in HCC cells with STOML2 high expression (Fig. 3f). Collectively, these results indicated the important roles of STOML2 on mitophagy, which might tightly connect with HCC proliferation and invasion.

### STOML2 regulates mitophagy by interacting with PINK1 and contributes to its stability

**Fig. 4:**
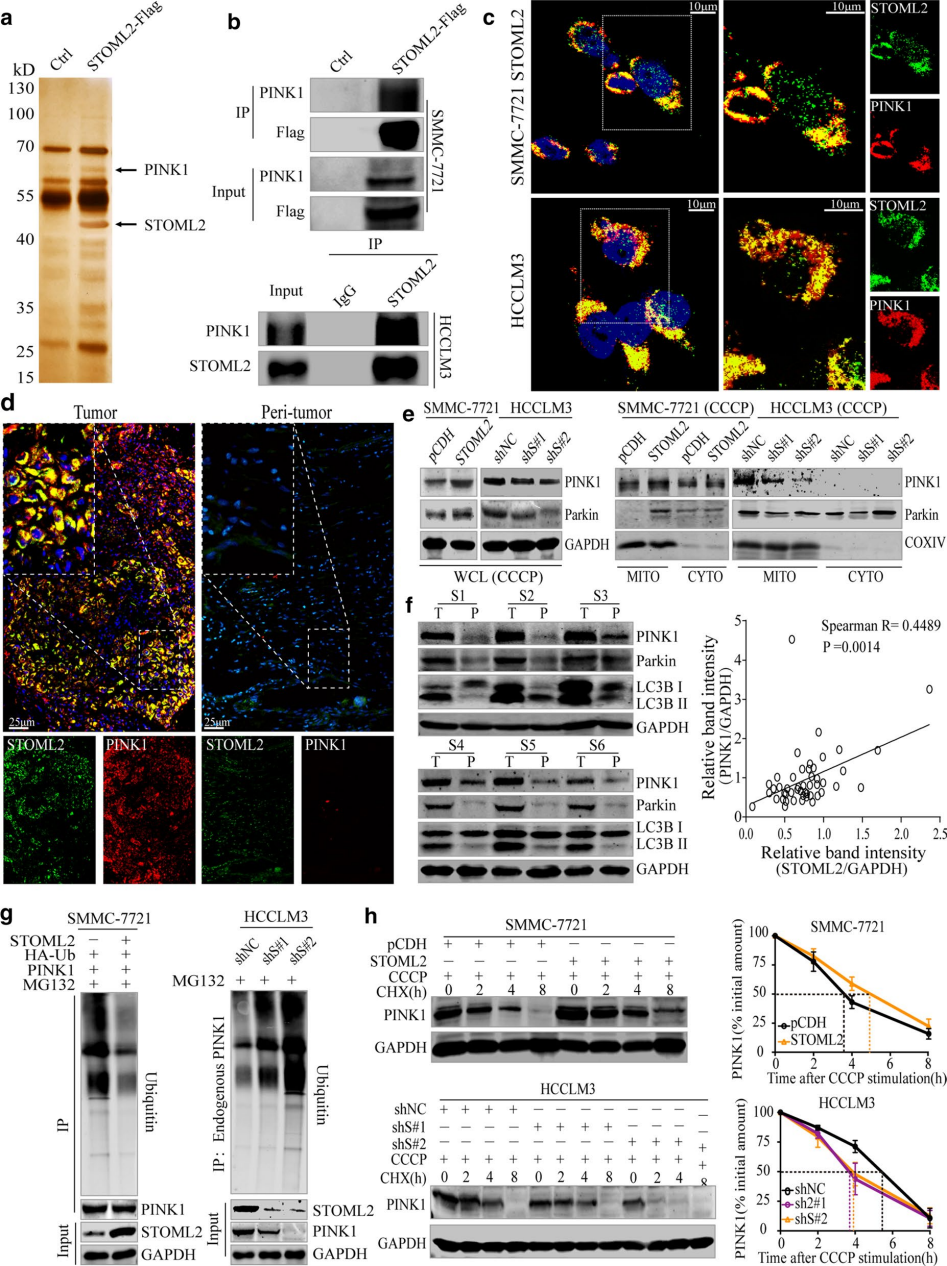
STOML2 interacted with PINK1 and contributed to its stability. **a** Total cell lysate was extracted from STOLM2 Flag-expressing or control cells treating with CCCP (10 μM), purified and resolved on SDS-PAGE. Silver stained gel showed differential bands, then the bands were retrieved and analyzed by MS. Identified PINK1 peptides are shown. **b** SMMC-7721 cells were transfected with STOML2-Flag or empty vector and subjected to immunoprecipitation using anti-Flag mAb. Co-immunoprecipitated PINK1 was detected using anti-PINK1 antibody (up panel). Endogenous STOML2 in HCCLM3 cells was immunoprecipitated using anti-STOML2 antibody with rabbit IgG as nonspecific control (down panel). Co-immunoprecipitated PINK1 was detected using anti-PINK1 antibody. **c** The co-localization between STOML2 (green) with PINK1 (red) was analyzed by confocal microscopy in SMMC-7721-STOML2 and HCCLM3 with CCCP (10 μM) stimulation. (Scale bar: 10 μm) **d** The expression and co-localization between STOML2 and PINK1 were analyzed in HCC and peri-tumor liver tissue of HCC patients by confocal microscopy. (Scale bar: 25 μm) **e** Under the treatment of CCCP (10 μM), total and mitochondrial protein levels of PINK1and Parkin were analyzed by Western blot. GAPDH and COX IV were used as loading controls. **f** Western blot analysis for PINK1, Parkin, LC3B I/II in 48 HCC tissues and peri-tumorous tissues patients (left panel), correlation analysis of the relative protein expression showed positive correlation between STOML2 and PINK1 in HCC patients (right panel). **g** Overexpression of STOML2 in SMMC-7721 increased accumulation of polyubiquitinated PINK1 when treated with MG132. PINK1 was pulled down and anti-ubiquitin antibody was used to detect polyubiquitinated PINK1 (left panel). Knockdown of STOML2 in HCCLM3 reduced polyubiquitinated PINK1 (right panel). **h** The upregulation of STOML2 reduces CCCP-induced PINK1 degradation. Western blot detected the alteration of PINK1 in SMMC-7721 and HCCLM3 with co-treatment of 10 μM CCCP and 10 μg/ml CHX for the indicated times (left panel). Densitometric analysis of PINK1 blots from three independent experiments is shown (right panel). GAPDH was used as a loading control

To uncover the underlying molecular mechanisms of STOML2 regulating HCC mitophagy, immunoprecipitation/mass spectrometry (IP/MS) was conducted to identify key molecules. STOML2-Flag produced in SMMC-7721 cells was immunoprecipitated by Flag mAb and coprecipitated proteins were visualized by sliver staining after electrophoresis and recognized by MS. One coprecipitated molecule turned out to be PINK1 (Fig. 4a), a key regulator of mitophagy. We next performed co-immunoprecipitation to verify whether STOML2 interacts with PINK1. PINK1 was precipitated by Flag-STOML2-tagged beads but not control beads in SMMC-7721 upon CCCP treatment, and the interaction between endogenous STOML2 with PINK1 was also confirmed in HCCLM3 (Fig. 4b). Furthermore, we observed the intracellular distribution of STOML2 and PINK1 in the presence of CCCP stimulation by confocal laser microscopy, which indicated that STOML2 co-localized with PINK1 in SMMC-7721-STOML2 and HCCLM3 (Fig. 4c). The similar co-localization was also found in STOML2 high expression HCC tissues (Fig. 4d). Furthermore, the expression and co-localization between PINK1 and COXIV were strong in SMMC-7721-STOML2 and HCCLM3 versus sparse expression of PINK1 and weak co-localization with COXIV in SMMC-7721, HCCLM3-shS#1 and shS#2 (Additional file 5: FigureS3A-B). Then, Western blot was carried out to detect the expression of STOML2, PINK1, and Parkin. The result showed that variation tendency of PINK1 and Parkin was consistent with STOML2, increased when STOML2 was upregulated, and vice versa, especially in the mitochondrial level (Fig. 4e). Notably, we detected and compared the expression of PINK1, Parkin, and LC3B I/II in HCC tissues and peri-tumor liver tissues (Fig. 4f). Identical to what have been found, the expression of PINK1, Parkin, and LC3B II in HCC tissues was much higher than that in peri-tumor liver tissues, and the statistical results showed positive correlation between STOML2 and PINK1 (Fig. 4f, right panel).

To determine the mechanism of the phenomenon that PINK1 could be upregulated by STOML2, qPCR analysis was carried out. We found that neither overexpression nor knockdown of STOML2 had little effect on transcription level of PINK1 (Additional file 5: Figure S3C), whereas the protein levels kept the similar variation tendency (Fig. 4e). The above results indicated that STOML2 upregulates PINK1 at the protein level rather than the mRNA level. Subsequently, we investigated the effect of STOML2 on the protein stability of PINK1. MG132, the proteasome-specific inhibitor, could protect PINK1 protein from degradation in STOML2-low expression HCC cells (Additional file 5: Figure S3D). A significant decrease of polyubiquitinated PINK1 protein was observed in STOML2-overexpression SMMC-7721 while STOML2-KD in HCCLM3 had the converse phenomenon (Fig. 4g). Furthermore, we tested the effect of STOML2 on PINK1 degradation rate by cycloheximide (CHX) pulse-chase assay, under the treatment of CCCP, STOML2-overexpression could dramatically prolong the half-life of PINK1 protein after adding CHX. In contrast, STOML2-KD remarkably decreased the half-life of PINK1 protein (Fig. 4h). These results indicated that STOML2 could inhibit the degradation of PINK1.

### Lenvatinib induces STOML2-dependent cytoprotective mitophagy in HCC cells

**Fig. 5:**
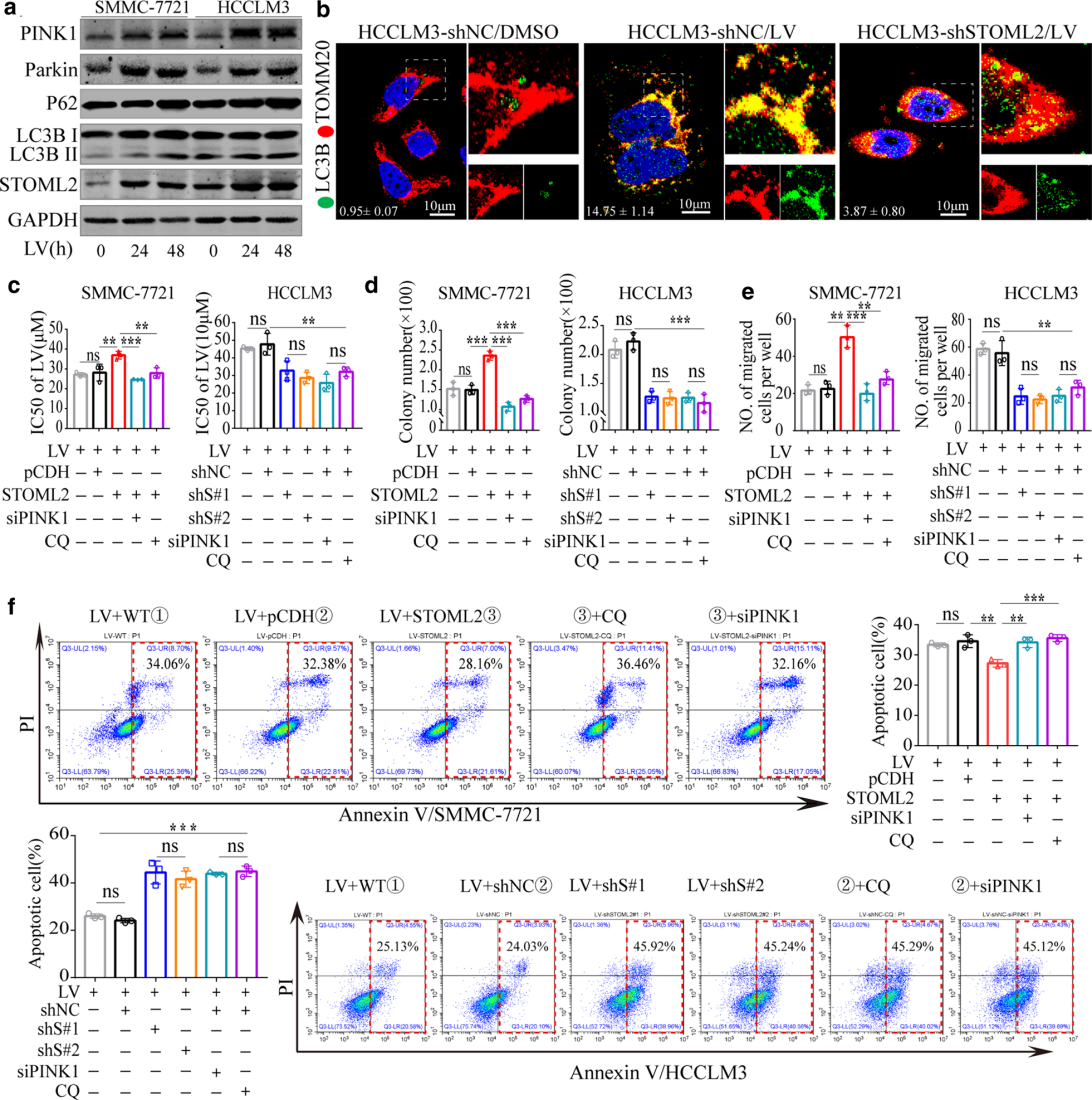
Inhibition of mitophagy sensitizes HCC cells to Lenvatinib treatment. **a** Western blot for PINK1, Parkin, p62, LC3BI/II in SMMC-7721 and HCCLM3 treated with lenvatinib (LV, 10 μM) at indicated time. GAPDH was used as a loading control. **b** The expression and co-localization of LC3B (green) with TOMM20 (red) increased sharply in HCCLM3-shNC cells treated with LV (10 μM) for 24 h, while the increase was counteracted significantly by shSTOML2 in HCCLM3 cells. The relative fluorescence intensity of LC3B is shown in the lower left corner. (Scale bars:10 μm) **c-f** SMMC-7721 and HCCLM3 control and derived cells were co-treated with LV(5 μM) and CQ (5 μM) or transiently transfected with PINK1 siRNA. IC50s of lenvatinib increased when STOML2 was upregulated (**c**). Identical to the IC50, under aforementioned conditions, colony formation (**d**) and migration (**e**) increased in HCC cells with STOML2 upregulation versus decreased with STOML2 downregulation. Correspondingly, apoptosis resulted from LV increased in HCC cells when STOML2 or PINK1 was downregulated or co-treated with CQ (**f**). **P < 0.01; ***P < 0.001; *ns* no significance, *LV* lenvatinib, *WT* wild type

It is well known that facing all kinds of stresses, such as antiangiogenesis, tumor has to change accordingly to develop drug insensitivity to survival. Increasing evidences suggest mitophagy is involved in the process. We postulated that lenvatinib, a first line antiangiogenesis drug for HCC, could cause mitochondrial dysfunction and induce mitophagy, which may be close correlated with the widespread drug insensitivity of antiangiogenesis. First, the mitochondrial membrane potential (MMP), a key inducing factor of mitophagy, was evaluated in SMMC-7721 and HCCLM3 with lenvatinib treated. JC-1 staining was applied to monitor MMP of cells by cytometry [24]. The measurement demonstrated that MMP of SMMC-7721 and HCCLM3 with lenvatinib treated decreased significantly compared with controls (Additional file 6: Figure S4A). Lenvatinib also promoted the transformation of LC3B I to LC3B II, with increased expression of PINK1, Parkin, and p62 in a time-dependent manner (Fig. 5a). Confocal co-localization analysis supported what have been demonstrated by Western blot. Compared with DMSO treatment, the co-localization between PINK1 and COXIV increased sharply as lenvatinib treated in HCCLM3-shNC cells (Additional file 6: Figure S4B). More LC3B, LAMP1 were detected and co-localized with mitochondria marker for the most part in lenvatinib treated cells (Fig. 5b, Additional file 6: Figure S4C). Furthermore, STOML2-KD in HCCLM3 partly counteracted the enhancement of PINK1 and LC3B with lenvatinib treatment. The co-localization between LC3B, PINK1, LAMP1, and mitochondrial marker also significantly decreased, respectively (Fig. 5b, Additional file 6: Figure S4B-C). In brief, these data indicated that mitophagy could be activated by lenvatinib, and STOML2 played important an role in mitophagy activation.

### Inhibition of STOML2-dependent mitophagy sensitizes SMMC-7721/HCCLM3 cells to Lenvatinib treatment

To explore the role of STOML2 on malignant potential in HCC cells following lenvatinib treatment, we compared the different responses to lenvatinib treatment in SMMC-7221, HCCLM3, and derived cells. STOML2-KD in HCCLM3 cells enhanced the cellular response to lenvatinib (Fig. 5c), inhibiting cell colony formation (Fig. 5d, Additional file 7: Figure S5A), migration (Fig. 5e, Additional file 7: Figure S5B) and inducing total apoptosis (Fig. 5f) to a greater extent, whereas overexpression of STOML2 in SMMC-7221 had the opposite effects (Fig. 5c–f). Notably, blockade of mitophagy by chloroquine (CQ) or PINK1 siRNA (Additional file 7: Figure S5C-D) reversed STOML2-related protection against lenvatinib (Fig. 5c–f, Additional file 7: Figure S5A-B). In conclusion, STOML2-induced mitophagy served as a protective function in HCC cells treated with lenvatinib, and blocking mitophagy enhanced the inhibition efficacy of lenvatinib to HCC cells.

### Lenvatinib increases the expression of STOML2 by upregulating hypoxia-inducible factor 1α, which transcriptionally regulates STOML2

**Fig. 6:**
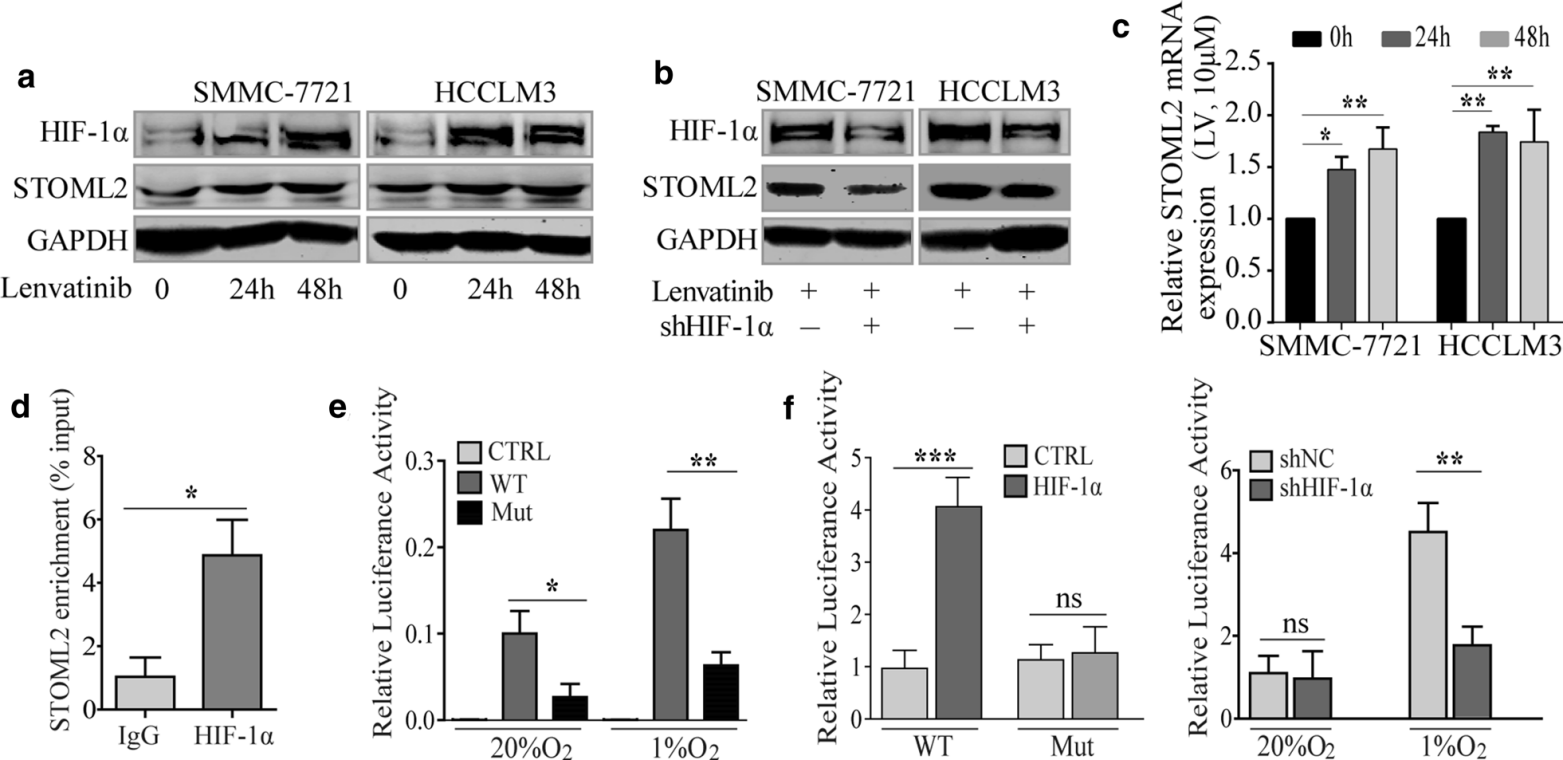
STOML2 is upregulated by Lenvatinib in HCC with HIF-1α dependent. **a** Western blot analysis of STOML2 and HIF-1α expression in both SMMC-7721 and HCCLM3 cells after exposure to lenvatinib (10 μM) for 24 h and 48 h. **b** Knockdown of HIF-1α expression in both SMMC-7721 and HCCLM3 cells counteracted the upregulation of STOML2 caused by lenvatinib. **c** qRT-PCR analysis of STOML2 expression in both SMMC-7721 and HCCLM3 cells after exposure to lenvatinib (10 μM) for 24 h and 48 h. **d** Endogenous HIF-1α associated with the STOML2 promoter. ChIP qRT-PCR assay was performed with sonicated chromatins immunoprecipitated from HCCLM3 cells by anti-HIF-1α mAb or preimmune IgG (control). **e** CTRL, WT and Mut reporter were exposed to 20% O_2_ or 1% O_2_ for 12 h in the SMMC-7721 cells. **f** Overexpression of HIF-1α stimulated the STOML2 promoter reporter activity in SMMC-7721 cells (left panel). Knockdown of HIF-1α expression downregulated the STOML2 promoter reporter activity in the HCCLM3 cells (right panel). *P < 0.05; **P < 0.01; ***P < 0.001; *ns* no significance, *Mut* mutation, *CTRL* control

Hypoxia is one of the main contributors to the acquisition of drug resistance and closely related to a worse prognosis [25, 26]. As one of the multi-kinase inhibitors, lenvatinib inhibits HCC growth and metastasis significantly, but accentuates hypoxia environment in tumor tissue. So we wonder whether hypoxia-inducible factor 1α (HIF-1α), a master regulator of oxygen homeostasis [27], could be regulated by lenvatinib. Thus, we investigated the expression of HIF-1α under the treatment of lenvatinib (10 μM) and Western blot analysis indicated that HIF-1α expression increased gradually with the time extending (Fig. 6a). Interestingly, we also found that STOML2 was increased simultaneously (Fig. 6a). To further determine whether increase of STOML2 in lenvatinib treatment is HIF-1α dependent, we examined STOML2 expression in HCC cells with HIF-1α downregulation and found that lenvatinib-induced STOML2 upregulation was abrogated upon silencing HIF-1α in both SMMC-7721 and HCCLM3 (Fig. 6b). An increase of STOML2 expression might result from activated STOML2 transcription and/or elevated STOML2 protein stability. Indeed, we found STOML2 mRNA levels were significantly increased upon lenvatinib treatment (Fig. 6c), suggesting that STOML2 might be transcriptionally regulated by HIF-1α. To confirm whether STOML2 is a direct transcriptional target of HIF-1α, STOML2 promoter region from − 2000 bp to the first ATG was cloned and putative hypoxia-responsive element (HRE) containing HIF-1α-binding consensus sequence 5′-A/GCGTG-3′ was located (Additional file 8: Figure S6A-B). To identify whether HIF-1α could bind at this site, chromatin immunoprecipitation (ChIP) assay was carried out and the results showed that the chromatin fragments from HCCLM3 cells immunoprecipitated by anti-HIF-1α mAb specifically enriched compared to IgG group (Fig. 6d). The sequences containing the putative 5′-promoter region of STOML2 (WT) or the region with mutated binding site (Mut) were cloned into luciferase promoter reporter vectors. The activity of wild type STOML2 promoter (WT) was significantly increased in hypoxic SMMC-7721 cells, while it was decreased in the HRE mutation group which further suggested the predicted HRE in the STOML2 promoter region is functional (Fig. 6e, Additional file 8: Figure S6B). In addition, over-expression of HIF-1α stimulated the STOML2 promoter reporter activity in SMMC-7721 cells while knockdown of HIF-1α expression downregulated the STOML2 promoter reporter activity in the HCCLM3 cells (Fig. 6f). Taken together, these data suggested that STOML2 was a direct transcriptional target of HIF-1α.

### Mitophagy inhibition enhances anti-tumor effect of lenvatinib in vivo

**Fig. 7:**
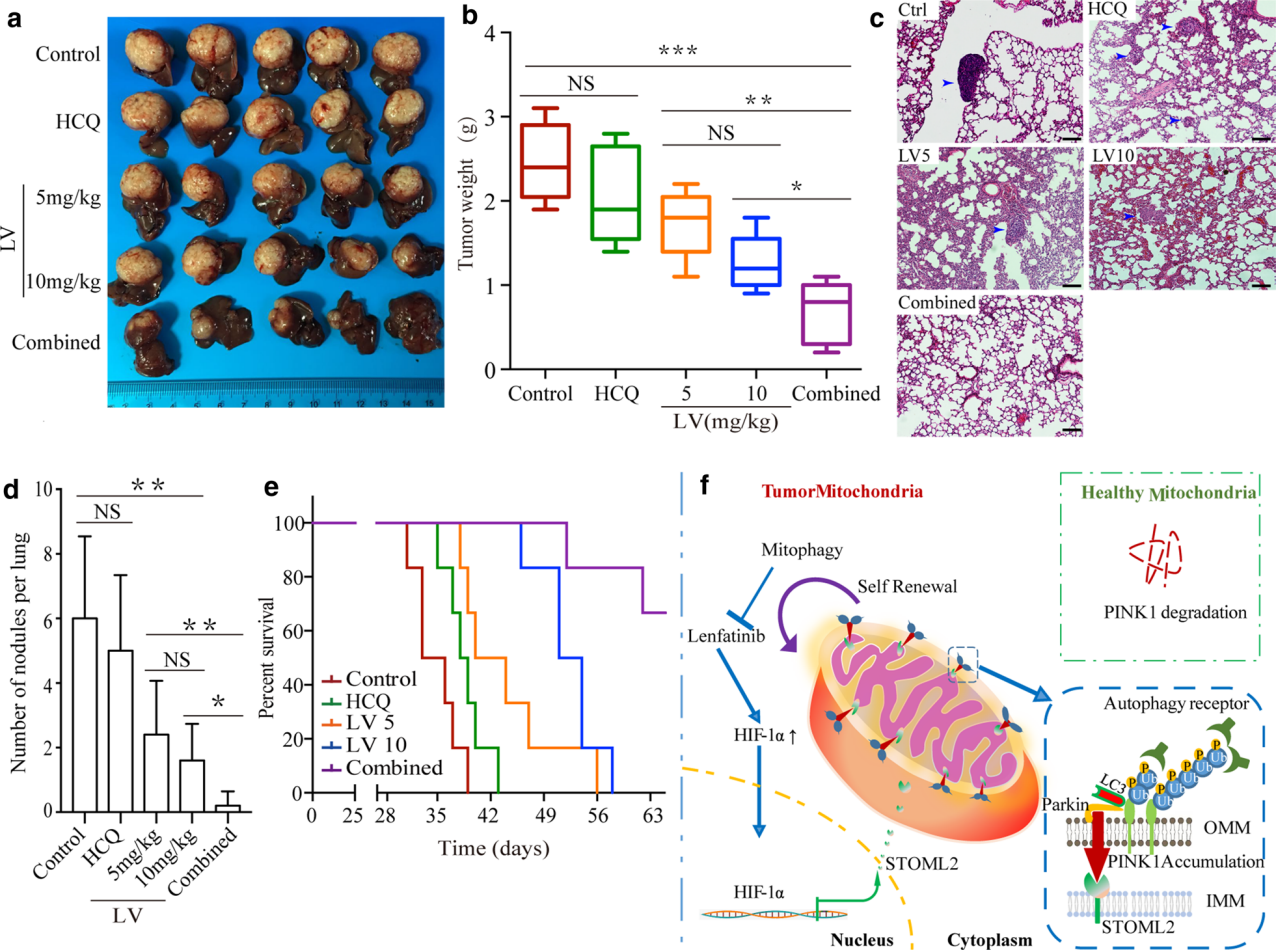
Inhibition effects of combination therapy with Lenvatinib and hydroxychloroquine were much better than Lenvatinib alone. **a–e **HCCLM3 orthotopically bearing mice were treated with HCQ, lenvatinib (5 mg/kg or 10 mg/kg) alone or combination with lenvatinib 5 mg/kg and HCQ 50 mg/kg, normal saline as control. **a** Representative images of the HCCLM3 orthotopic HCC tumors from each group (*n* = 5 mice/group). **b** Tumor weight in both low and high lenvatinib treatment groups was much lower than that in control group, while there was no difference between control and HCQ groups. The best inhibition efficacy was found in combination therapy group. **c** Representative H&E staining images of lung tissues of different treatment groups. Blue arrows indicated lung metastasis. Scale bar, 100 μm. **d** The mean number of lung metastasis in mice of each group. **e** The results of Kaplan–Meier survival curves for tumor bearing mice indicated that combination treatment prolonged OS of xenograft mice models bearing HCCLM3 significantly (*P* < 0.001), even much better than high dose of lenvatinib treatment group (*P* = 0.006). The *P* values for each comparison are as followed: Control versus HCQ (*P* = 0.065), Control versus LV5 (*P* = 0.023), Control versus LV10 (*P* < 0.001). **f** Schematic depiction of the underlying mechanisms of STOML2 upregulation in HCC and its functional role of facilitating HCC proliferation, metastasis and drug insensitivity via promoting PINK1-Parkin-mediated mitophagy. *P < 0.05; **P < 0.01; ***P < 0.001; *ns* no significance, *LV5* lenvatinib 5 mg/kg, *LV10* lenvatinib 10 mg/kg

To confirm these in vitro findings, we further examine the efficacy of lenvatinib (5 mg/kg or 10 mg/kg, respectively) and hydroxychloroquine (50 mg/kg) alone, or combination (lenvatinib 5 mg/kg + hydroxychloroquine 50 mg/kg) on HCC growth and metastasis in immunodeficient mice bearing orthotopic HCCLM3 xenograft tumors compared to normal saline control. As shown in Fig. 7, statistical differences were found among control, lenvatinib low and high dose groups. When compared with the tumor weight in control group (2.52 ± 0.42 g), lenvatinib reduced tumor weight in a dose-dependent mode, 1.74 ± 0.41 g for 5 mg/kg and 1.26 ± 0.33 g for 10 mg/kg, respectively (Fig. 7a, b), whereas the tumor inhibition effects were limited in hydroxychloroquine treatment group (2.06 ± 0.587 g). The similar effects on lung metastasis inhibition and prolonged survival were also found in those groups received lenvatinib treatment. Notably, the highest suppression of primary tumor growth was found in mice treated with the combination therapy. The average tumor size was 0.68 ± 0.37 g, with tumor growth inhibition rate 73%. The lowest lung metastasis was also achieved in this group (Fig. 7c, d). Additionally, the median overall survival of mice increased from 36 days in control group to over 63 days (*P* < 0.001) in combination therapy group. The effect of the combination treatment group is even better than high dose of lenvatinib treatment group (*P* = 0.006) (Fig. 7e).

Taken together, these in vitro and in vivo gain- and loss-of-functional studies and in vivo combination therapy demonstrated that STOML2 played important roles in mitophagy, which resulting in HCC growth and invasion. Lenvatinib and chloroquine/ hydroxychloroquine combination treatment inhibited the growth and metastasis of HCC and increased median OS. Schematic representation of the major molecular mechanism was shown in Fig. 7f.

## Discussion

The flexibility of mitochondria is conducive to survival of HCC cells in confront of adverse environmental conditions such as hypoxia, starvation, and persistent chemotherapeutic and targeted therapy [2]. Mitophagy is involved in the process of eliminating damaged mitochondria. Increasing evidences suggest that mitophagy is crucial for cancer growth and metastasis [28]. However, a more in-depth understanding of factors regulating mitophagy in HCC development is urgently needed, especially the study of mitochondrial proteins in the process. STOML2, an inner mitochondrial membrane protein, has been demonstrated promoting cancer development in several cancers [29–31]. With genome-wide profiling analysis, we found STOML2 is one of the major upregulated genes in HCC with extrahepatic metastasis when compared with HCC without metastasis [17]. In the present study, we show that HCC with high STOML2 expression has more malignant potential and poor prognosis. Mechanistically, STOML2 may trigger cytoprotective mitophagy via interacting and stabilizing PINK1 in HCC cells under cellular stress. Furthermore, we found STOML2 was upregulated with HIF1 α-dependent in HCC cells treated with lenvatinib, which may promote HCC insensitivity to lenvatinib treatment. Combination of lenvatinib and chloroquine/ hydroxychloroquine that concurrently block both angiogenesis and mitophagy that are upregulated in response lenvatinib further improve the curative efficacy. Collectively, we pinpointed STOML2 for the first time as a critical factor that promoted HCC metastasis and insensitivity to antiangiogenesis drugs through regulating mitophagy.

Mitophagy-induced mitochondrial removal is a response to mitochondrial injury allowing for cellular adaptation to the microenvironment stresses. Dysregulation of the process is known to be an important mediator of tumor progression [32]. In this study, through gene expression profiling in clinical HCC tissues, gain- and loss-of-functional validation in HCC cell lines, we have demonstrated STOML2 as an independent prognostic predictor, played vital roles in promoting HCC growth and invasion. Growing number of evidences showed that STOML2 is closely related to higher malignant potential in a variety of tumors [30, 31]. So far the study of STOML2 in cancer progression is still remaining on the stage of observation.

One interesting question is how STOML2 promotes HCC growth under stresses. Analysis of co-immunoprecipitation disclosed that the interaction between STOML2 and mitochondrial kinase PINK1 played a critical role in PINK1-Parkin-mediated mitophagy, through which it promoted HCC growth, metastasis. The stabilization of PINK1 on the membrane of mitochondria is a critical factor that regulates activation of Parkin-mediated mitophagy. We first demonstrated that overexpression of STOML2 promoted the accumulation of PINK1 on the mitochondrial membrane with longer half-life and subsequently initiated PINK1-Parkin-mediated mitophagy. Conversely, STOML2-KD significantly decreases the half-life of PINK1. These results suggested that STOML2 regulated the stability of PINK1 through a direct interaction with this protein and served as a novel important regulator of the PINK1-Parkin system.

As reported before, the role of PINK1-Parkin-mediated mitophagy in the regulation of cell death is debated. Is mitophagy beneficial or harmful to cancer? The results depend on the context to a great extent. Generally, for tumorigenesis decreased mitophagy may allow for the persistent of dysfunctional mitochondria or tumorigenic mitochondrial signals, whereas for established tumors mitophagy may be required for stress adaptation and survival [32, 33]. Supporting this concept, PINK1 expression has been reported to be upregulated in lung cancer, which promotes the proliferation and chemoresistance [34]. It is also reported that PINK1 and LC3 were significantly upregulated in the esophageal squamous cell carcinoma patients, and inhibition of mitophagy restored the chemosensitivity in those patients [35]. In this study, the stability of PINK1 was enhanced significantly in HCC cells when STOML2 was upregulated. The malignant potential, especially for migration and invasion, increased in those HCC cells, which was further supported by the results of in vivo study. Furthermore, the enhancement of malignant potential in HCC cells was inhibited significantly when treated with autophagy inhibitor or downregulation PINK1.

As one of the first line treatment drugs, lenvatinib is widely used and prolongs the OS of advanced HCC patients [36]. However, the objective response rate in REFLECT trial and real-world study is usually less than 30% judged by Response Evaluation Criteria in Solid Tumors (RECIST) 1.1. largely because of some HCC patients are not sensitive to lenvatinib treatment, which deserves further study eagerly [37]. Indisputably, antiangiogenesis results in severe hypoxia in tumor environment. Our results demonstrated that HIF-1α increased sharply in HCC cells treated with lenvatinib, binding to HRE of STOML2 promoter, and thus transcriptionally promoted the expression of STOML2. Meanwhile, the ratio of LC3B II/LC3B I and PINK1 increased remarkably with more co-localization between LC3B, PINK1, LAMP1, and mitochondria marker, respectively. These fundings suggested mitophagy is activated by lenvatinib.

More interestingly, knockdown of STOML2 did display the restriction of PINK1-Parkin-mediated mitophagy and increased sensitivity to lenvatinib in HCC cells. Inhibiting mitophagy by PINK1 siRNA or chloroquine also enhanced the inhibitory effects of lenvatinib on colony formation and invasion in STOML2-high expression cells. To further demonstrated what have been found in vitro study, the efficacy of combination treatment with lenvatinib and hydroxychloroquine was detected in immunodeficient mice bearing orthotopic HCCLM3 xenograft tumors. Whereas lenvatinib alone had a significant impact on HCC growth, the highest suppression of primary tumor growth and lung metastasis was found in combination treatment group, much better than lenvatinib or hydroxychloroquine treatment alone.

In summary, STOML2 was upregulated in HCC and correlated with poor prognosis in patients. In addition, our study provided a better understanding in both functional role and mechanism of STOML2 in HCC growth and metastasis. Notably, our results suggested for the first time that lenvatinib-induced or hypoxia-induced HIF-1α could bind to HRE of STOML2 promoter and transcriptionally promoted the expression of STOML2. The upregulation of STOML2 could favor cyto-protective mitophagy via stabilizing PINK1 to facilitate cancer cell migration and invasion, relieve cellular stress, and regulate the sensitivity of HCC cells to lenvatinib. A molecular link between aberrant STOML2 and HIF-1α expression and their regulation of susceptibility to lenvatinib treatment in HCC was demonstrated in this study. STOML2 may become a prognostic marker and therapeutic target for HCC. Designing inhibitors targeting STOML2 or mitophagy is a promising approach to combine with antiangiogenesis for better curative efficacy.

## Conclusions

Collectively, our findings demonstrate that STOML2 could amplify mitophagy through interacting and stabilizing PINK1, which promote HCC metastasis and modulate the response of HCC to lenvatinib. Combinations of pharmacologic inhibitors that concurrently block both angiogenesis and mitophagy that are upregulated in response to antiangiogenesis may be effective treatments for HCC.

## Supplementary information

**Additional file 1:** Supplementary Materials and Methods.

**Additional file 2:** Supplementary Tables.

**Additional file 3: Figure S1.** STOML2 expression is upregulated in HCC tissues and predicts a poor prognosis. (**A**) The mRNA expression level of STOML2 in HCC tissues (T, n=369) compared with the normal liver tissues (N, n=50) by the GEPIA database. (**B-C**) Patients with high STOML2 expression have poorer overall survival and disease free survival compared with patients with low STOML2 expression based on the RNA sequencing expression data (group cutoff in 28%/72%) from the TCGA project. *P<0.05. *TPM* transcripts per million, *LIHC* Liver hepatocellular carcinoma, *HR* hazard ratio.

**Additional file 4: Figure S2.** STOML2 expression is upregulated in HCC cells, promotes HCC proliferation and inhibits apoptosis in vitro. (**A**–**B**) Expression of mRNA (**A**) and proteins (**B**) levels of STOML2 in different HCC cell lines. Significantly increased STOML2 levels were detected in HCC cell lines especially in those with higher invasive and metastatic capabilities cells (MHCC-97H and HCCLM3) compared with L02. (**C**–**D**) The effects of STOML2 gain- or loss-of-function on in vitro proliferation (**C**) and apoptosis (**D**) by colony formation assay and flow cytometric analysis. *P<0.05; **P<0.01; ***P<0.001; *ns*, no significance.

**Additional file 5: Figure S3.** STOML2 promotes mitophagy in HCC cells under stress. (**A**–**B**) Confocal microscopy was performed to detect spatial colocalization of mitochondrial protein COXIV (red) and PINK1 (green) in SMMC-7721 and HCCLM3 control and derived cells under the treatment of CCCP (10 μM) for 4 h. (Scale bars:10 μm) (**C**) mRNA levels of STOML2 and PINK1 were determined by qRT-PCR in SMMC-7721 and HCCLM3 with manipulated the expression of STOML2, taking GAPDH mRNA as a control. (**D**) The alteration of PINK1 protein in SMMC-7721 and HCCLM3 control and derived cells with co-treatment of 10μM CCCP and 20 μM MG132 was detected by Western blot. **P<0.01; ***P<0.001; *ns*, no significance.

**Additional file 6: Figure S4.** Lenvatinib induces cytoprotective mitophagy in HCC cells. (**A**) Double fluorescence staining of mitochondria by JC-1 was applied to monitor the mitochondrial membrane potential (MMP), as green fluorescent J-monomers indicating loss of MMP, and red fluorescent J-aggregates reflecting higher MMP. The MMP of SMMC-7721 and HCCLM3 with lenvatinib (10 μM, 4 h) treatment decreased significantly compared with controls by flow cytometric analysis. (**B-C**) Confocal microscopy was performed to detect spatial colocalization of mitochondrial protein COXIV (red) and PINK1 (green) (**B**) or TOMM20 (red) and LAMP1 (green) (Scale bars:10 μm) (**C**) in HCCLM3 cells with or without the treatment of lenvatinib (10 μM, 24 h). (Scale bars:10 μm) **P<0.01; ***P<0.001; *ns*, no significance.

**Additional file 7: Figure S5.** Inhibition of mitophagy suppresses HCC migration and sensitizes HCC cells to Lenvatinib treatment. (**A**–**B**) SMMC-7721 and HCCLM3 control and derived cells were co-treated with LV (5μM) and CQ (5μM) or transiently transfected with PINK1 siRNA. In STOML2-high expression cells, the effect of lenvatinib on inhibiting colony formation (**A**) and migration (**B**) of HCC cells was weakened while the inhibitory effect of lenvatinib was restored in when CQ treated or siPINK1 transfected cells. (**C**–**D**) Silencing PINK1 with short interfering RNA in SMMC-7721-STOML2 and HCCLM3. **P<0.01; ***P<0.001.

**Additional file 8: Figure S6.** STOML2 is a target of HIF-1α. (**A**) HRE in the STOML2 promoter identified by the JASPAR database (http://jaspar.genereg.net/). (**B**) Schematic illustration of STOML2 promoter region with potential HIF-1α binding site. The WT and HRE mutant sequences were indicated.

## Data Availability

All data generated or analyzed during this study are included either in this article or in the supplementary Materials and Methods, Tables, Figures, and Figure Legends files.

## Abbreviations

HCC: Hepatocellular carcinoma
mRNA: Messenger RNA
qRT-PCR: Quantitative real-time PCR
IP/MS: Immunoprecipitation/mass spectrometry
mAb: Monoclonal antibody
OS: Overall survival
TMA: Tissue microarray
TTR: Time to tumor recurrence
STOML2: Stomatin-like protein 2
shNC: Negative control shRNA
shS#1: Short hairpin STOML2 #1
shS#2: Short hairpin STOML2 #2
GAPDH: Glyceraldehyde-3-phosphate dehydrogenase
MDC: Monodansylcadaverine
PINK1: PTEN-induced putative kinase 1
CCCP: Carbonyl cyanide mchlorophenylhydrazone
VDAC1: Voltage-dependent anion channel 1
TOMM20: Translocase of outer mitochondrial membrane 20
COXIV: Cytochrome c oxidase IV
LAMP1: Lysosomal-associated membrane protein 1
CHX: Cycloheximide
CQ: Chloroquine
HCQ: Hydroxychloroquine
HIF-1α: Hypoxia-inducible factor 1α
ChIP: Chromatin immunoprecipitation
H&E Staining: Hematoxylin–eosin staining
