# Supplementary material for: STOML2 potentiates metastasis of hepatocellular carcinoma by promoting PINK1-mediated mitophagy and regulates sensitivity to lenvatinib
Source: J Hematol Oncol. 2021 Jan 14;14:16. doi: 10.1186/s13045-020-01029-3 (PMC7807703; doi:10.1186/s13045-020-01029-3)
Supplement: Supplementary file 1 — Additional file 1: Supplementary Materials and Methods. [file 13045_2020_1029_MOESM1_ESM.docx]

**Supplementary Materials and Methods**

**Clinical samples**

The first set of HCC samples were obtained from patients who underwent surgical resection at Department of General Surgery, Huashan Hospital, Fudan University (Shanghai, China). The second set of HCC samples were obtained from Liver Cancer Institute, Zhongshan Hospital, Fudan University (Shanghai, China). Follow-up of the second set patients was completed with the longest follow-up of 60 months. As described in previous study, Hepatitis B history was defined as history with positive serum hepatitis B surface antigen, tumor with complete encapsulation was defined as tumor surrounded by a fibrous capsule or a thinner pseudocapsule continuously. Tumor differentiation was graded by Edmondson grading system. Vascular invasion was defined as the presence of tumor within a vascular lumen lined by endothelium under microscopy. Laboratory tests including serum α-fetoprotein (AFP) and alanine aminotransferase (ALT), abdominal ultrasonography and chest radiography were performed every 2-6 months after operation while enhancement computed tomography scanning or magnetic resonance imaging was performed every 6-12 months or suspicious recurrence. Treatment after recurrence was administered according to the guideline. Overall survival (OS) was defined as the interval between surgery and either death or the last observation taken. Time to tumor recurrence (TTR) was defined as the interval between surgery and recurrence.

**Cell culture and reagents**

The human HCC cell lines SMMC-7721 and HCCLM3 were obtained from the Cell Bank of the Chinese Academy of Sciences and cultured in Dulbecco’s modified Eagle’s medium (DMEM; HyClone, SH30243.01) supplemented with 10% FBS (Gibco, 10099141) 100 U/mL penicillin and 100 mg/mL streptomycin (Invitrogen, 15070063) at 37°C with 5% CO_2_. Short tandem repeats (STR) analysis of SMMC-7721 was carried out. NEPCO 1000 hypoxia incubator (Warrensburg, NY) was applied to Hypoxia (1% O_2_) experiments. Carbonyl cyanide 3-chloro-phenylhydrazone (CCCP, C2759) and hydroxychloroquine (HCQ, 5092720001) were purchased from Sigma-Aldrich. Cycloheximide (CHX; 2212S) and chloroquine (CQ; 14774S) were purchased from Cell Signaling Technology while lenvatinib (S1164) and MG132 (S2619) were purchased from Selleck.

**RNA extraction, reverse transcription and quantitative real-time polymerase chain reaction (qRT-PCR)**

Total RNA was isolated from HCC tissues and cell lines using TRIzol RNA isolation reagent (Invitrogen, 15596026) and reverse transcribed using a PrimeScript™ RT reagent kit (Takara, RR037A) according to the manufacturer’s instructions. QRT-PCR was performed with an SYBR Premix EX Taq™ II kit (Takara, RR820A) using Light Cycler 480 system (Roche Diagnostics, Germany). The respective primers (BioTeZ, Berlin, Germany) used are shown in Supplementary Table 2. Relative genes expression analysis was performed using the eq. 2 ^-△△CT^, with GAPDH used as an internal control.

**Western blotting**

Cells were lysed in RIPA (Beyotime, P0013B) containing protease inhibitor phenylmethanesulfonyl fluoride (PMSF) (Beyotime, ST506). Protein was denatured at 100 °C for 15 min, electrophoretically separated on SDS–PAGE and then transferred onto a nitrocellulose membrane (Millipore, HATF00010). The membrane was blocked in 5% skim milk for 60 min at room temperature (RT), incubated overnight with a primary antibody at 4 °C. After being washed three times in 30 min, the membrane was incubated with a secondary antibody with DyLight fluorescent dye labeled for 1 h at RT, washed three times in 30 min. The membranes were scanned by an Odyssey Imaging System (LI-COR Biosciences, USA). Antibodies are listed in Supplementary Table 1.

**Immunohistochemical (IHC) staining**

Briefly, after deparaffinized with xylene, rehydrated in graded ethanol, immersed in 0.3% hydrogen peroxide, and heat-mediated antigen retrieval in citric acid at pH 6.0, tissue sections were incubated with the antibody for STOML2 at 4 °C overnight, labeled by HRP (rabbit) second antibody at room temperature for 60min. Finally, sections were developed in DAB (DAKO, GK500705) solution under microscopic observation and counterstained with hematoxylin. Immunohistochemical scoring criteria are detailed in Supplementary material. A proportion score, which represents the estimated proportion of positively stained tumor cells, was assigned as follows: < 10%, 0; 10 to 25%, 1; 26 to 50%, 2; 51 to 75%, 3; and > 75%, 4. An intensity score, which represents the average intensity of the positive tumor cells, was assigned as follows: 0 (no staining), 1 (intensity lower than positive control), 2 (intensity equal to positive control), 3 (intensity higher than positive control). The proportion and intensity scores were then multiplied to obtain a total score, which ranged from 0 to 12. A total score of 0, 1 to 4, 6 to 8, and 9 to 12 was defined as being negative (–), weak positive (+), moderate positive (++), and strong positive (+++), respectively. The final scores were designated as low or high expression as follows: low expression (negative and weak positive), high expression (moderate positive, strong positive).

**Establishment of overexpression or knock-down cell lines**

For overexpression of STOML2 in HCC cell lines, the full-length cDNA encoding STOML2 was subcloned into the pCDH-CMV-MCS-EF1-Puro vector. After virus packaged in 293 T cells, STOML2 or mock vectors were transfected into SMMC-7721. For STOML2-knockdown cell lines, shRNA sequences were cloned into a pLKO.1-TRC cloning vector, target sequences are shown in Supplementary Table 2. shHIF1α (sc35561) and PINK1 short interfering RNA (siRNA, sc44598) were purchased from Santa Cruz Biotechnology.

**MTT assay**

The cancer cells were seeded in 96-well plates at a density of 2 × 10^3^ cells per well. Cells were treated with indicated regents or not. Cell viability was measured using the Cell Counting Kit (CCK8; Beyotime, C0043) according to the manufacturer’s instructions at specified time points with a multifunctional microplate reader (Bio-Rad Laboratories, Hercules, CA).

**Colony formation assay**

Five hundred cells were seeds in 24-wells plate per well. After cultured for 7 days, we washed the plate with PBS for two times and fixed cells with 4 % Paraformaldehyde (Servicebio, G1101) for 15 min, incubated cells with Crystal Violet Staining Solution (Beyotime, C0121) for 15 min and washed the staining solution with distilled water.

**Migration and invasion assay**

The migratory or invasive abilities of cells were evaluated by 24-well transwell chambers coated with or without Matrigel (BD Pharmingen, 356234). Briefly, 200 μl of the single cell suspension (5 × 10^4^ cells per well) were seeded in the upper chamber of the 8 μm-pore transwell filters (Corning, Costar 3422) with serum-free DMEM, and the lower chambers were supplemented with 800 μl DMEM containing 10% FBS. After incubation for 48-72 h, sterile cotton swabs were used to remove the cells remaining in the membrane of upper transwell chamber. Then the transwell chambers were fixed with 4 % Paraformaldehyde (Servicebio, G1101) and stained with Crystal Violet Staining Solution (Beyotime, C0121). The cells that migrated to the bottom side of the membrane were photographed and counted in 3 random fields by an inverted microscope (Nikon, Japan) at 200× magnification.

**Apoptosis study**

The Annexin V-FITC Apoptosis Detection Kit (BD 556547) was purchased from BD Pharmingen™ and used as recommended by manufacturer’s instruction. And the cell suspension was subsequently analyzed by flow cytometry (CytoFLEX, Beckman).

Immunohistochemical Ki-67 staining

The specific experimental process is similar to immunohistochemistry. The proliferation index was determined by Ki-67 immunostaining and calculating the number of 3,3’-diaminobenzidine–positive cells per total number of cells (hematoxylin-positive plus 3,3’-diaminobenzidine–positive cells) in 3 randomly selected fields at ×200.

**TUNEL Assay**

TUNEL assay was performed using TUNEL Assay kit- BrdU-Red (Abcam, ab66110) according to the manufacturer’s protocol.

**TCGA data collection and analysis**

Gene expression data for 372 cancer specimens profiled by TCGA were stratified in three groups according to STOML2 gene expression (low, intermediate, high). The 25th and 75th percentiles were used as cutoff thresholds. Differential expression of genes (DEGs) between low and high STOML2 expression was tested by two-tail student’s t test. Metascape (https://metascape.org/gp/index.html) was used to perform functional annotation of the DEGs.

**Flow Cytometry Detection of JC-1 Fluorescence**

Mitochondrial membrane potential assay kit with JC-1 (C2006) was purchased from Beyotime and used as recommended by manufacturer’s instruction.

**Histological Immunofluorescence**

The specific experimental process of histological Immunofluorescence is similar to IHC staining. STOML2 (Human Protein Atlas, HPA062016) and PINK1 (Abcam, ab186303) antibodies were used.

**Mitochondria isolation**

The Mitochondria isolation Kit (C3601) was purchased from Beyotime and used as recommended by manufacturer’s instruction.

**Transmission electron microscopy**

Cells were fixed with 2.5% glutaraldehyde and 1% osmium tetroxide, dehydrated with different concentrations of acetone, polymerized with epoxy resin and cut into ultrathin sections. After staining with aqueous uranyl acetate and lead citrate, t sections were observed by Hitachi TEM system.

**Immunoprecipitation and Immunblot (CO-IP).**

Whole cell lysates of HCC cells were incubated with Flag antibody or antibody against STOML2 and protein A/G agarose beads overnight, after 5 times washing with RIPA buffer, the proteins coupled to the beads were eluted in protein loading buffer and boiled, followed by Western blot to detect proteins with specific antibodies.

**Chromatin immunoprecipitation assay.**

HCCLM3 cells were cross-linked with 1% formaldehyde and reversed with 0.125 M glycine. After washing with PBS buffer, cells were collected and suspended in nuclei lyses buffer. Next, DNA was crushed into fragments by sonication. Anti-HIF-1α antibody or rabbit IgG with protein A/G-agarose beads were added and incubated overnight at 4 °C to immunoprecipitate DNA-containing complexes. DNA was isolated and used for PCR analysis.

**Luciferase reporter assay**

SMMC-7721 cells were seeded in a 24-well plate at 70% to 80% confluence. After 24 hours, cells were co-transfected with wild-type or mutant HIF-1α and PRL-TK reporter construct and then collected 48 hours after transfection. Luciferase activity was detected according to the manufacturer's instructions (Promega, E1910) by Microplate Spectrophotometer (Bio-Rad, Hercules, CA, USA).

**Tumor xenografts in nude mice**

Mice (BALB/c) were housed five mice per cage in a specific pathogen-free room with a 12 hours light/dark schedules at 25 ℃±1 ℃ and were fed an autoclaved chow diet and water ad libitum. In subcutaneous implantation nude mice models, tumor growth was monitored every 4 days and tumor volumes were calculated as (a × b^2^) / 2, a and b were the longest and shortest diameters of the tumors. In tail vein injection models, lung metastases were analyzed. Whole lungs were fixed with 4% paraformaldehyde before dehydration and embedding in paraffin. Consecutive sections made from every lung tissue Paraffin, sections were stained with H&E according to standard protocols and metastatic nodules were counted with a microscopic count assay. Survival was determining using Kaplan-Meier curve.
