# Supplementary material for: STOML2 potentiates metastasis of hepatocellular carcinoma by promoting PINK1-mediated mitophagy and regulates sensitivity to lenvatinib
Source: J Hematol Oncol. 2021 Jan 14;14:16. doi: 10.1186/s13045-020-01029-3 (PMC7807703; doi:10.1186/s13045-020-01029-3)
Supplement: Supplementary file 2 — Additional file 2: Supplementary Tables. [file 13045_2020_1029_MOESM2_ESM.docx]

| **Supplementary Table 1. Summary of primary antibodies used in Western Blot, Immunochemistry,** **immunofluorescence, Co-immunoprecipitation and Chromatin immunoprecipitation** | | | | | | | |
| --- | --- | --- | --- | --- | --- | --- | --- |
| Antibody | Concentration | | | | | Cat. No. | Company |
|  | for WB | for IHC | for IF | for co-IP | for CHIP |  |  |
| STOML2 | / | 1: 200 | / | / | / | HPA062016 | Human Protein Atlas |
| STOML2 | 1:1000 | / | 1:200 | / | / | #73956 | Cell Signaling Technology |
| STOML2 | / | / | / | 1:50 | / | ab191884 | Abcam |
| Ki-67 | / | 1: 100 | / | / | / | ab16667 | Abcam |
| PINK1 | 1:1000 | / | 1:100 | / | / | ab216144 | Abcam |
| Parkin | 1:1000 | / | / | / | / | #4211 | Cell Signaling Technology |
| p62 | 1:1000 | 1:100 | / | / | / | ab56416 | Abcam |
| LC3B | 1:1000 | / | 1:200 | / | / | #2775 | Cell Signaling Technology |
| COXIV | 1:1000 | / | 1:150 | / | / | #4844 | Abcam |
| Tim23 | 1:1000 | / | / | / | / | 11123-1-AP | Proteintech |
| VDAC1 | 1:1000 | / | / | / | / | 10866-1-AP | Proteintech |
| HIF1α | 1:1000 | / | / | / | 1:100 | # 36169 | Cell Signaling Technology |
| GAPDH | 1:3000 | / | / | / | / | ab9484 | Abcam |
| TOMM20 | 1:1000 | / | / | / | / | WH0009804M1 | Sigma-Aldich |
| COXIV | / | / | 1:200 | / | / | # 11967 | Cell Signaling Technology |
| LAMP1 | / | / | 1:200 | / | / | #4844 | Cell Signaling Technology |
| PINK1 | / | / | 1:100 | / | / | ab186303 | Abcam |
| Flag | 1:1000 | / | / | 1:50 | / | F7425 | Sigma-Aldich |
| normal rat IgG | / | / | / | 1:50 | / | I5006 | Sigma-Aldich |
| Anti-rabbit IgG (H+L) (DyLight™ 680 Conjugate) | 1:15000 | / | / | / | / | #5366 | Cell Signaling Technology |
| Anti-mouse IgG (H+L) (DyLight™ 800 4X PEG Conjugate) | 1:15000 | / | / | / | / | #5257 | Cell Signaling Technology |
| Goat Anti-Rabbit IgG H&L (Alexa Fluor® 488) | / | / | 1:500 | / | / | ab150077 | Abcam |
| Goat Anti-Mouse IgG H&L (Alexa Fluor® 647) | / | / | 1:500 | / | / | ab150115 | Abcam |
| Abbreviation: Co-IP, immunoprecipitation; WB, western blot; IHC, immunohistochemistry; IF, immunofluorescence; ChIP, Chromatin immunoprecipitation. | | | | | | | |
| Note: Cambridge, UK; Cell signaling Technology, Danvers, MA, USA; Proteintech Group, Inc, Pearl Street, Rosemont, USA; Sigma-Aldrich, Spruce Street, St. Louis, USA; Life Technology, Staley Road Grand Island, NY, USA. | | | | | | | |

**Supplementary Table 2. Primers and target sequences in this study**

| Identifier | Forward (5’-3’) | Reverse (5’-3’) |
| --- | --- | --- |
| **For qPCR** | | |
| STOML2 | CCCGCTGCAAGTATGATGG | GTTCCTGAGACGCTGTTC |
| PINK1 | CCGCTGCAAGTATGATGG | GTTCCTGAGACGCTGTTC |
| GAPDH | TGCGAGTACTCAACACCAACA | GCATATCTTCGGCCCACA |
| **For STOML2-Knockdown** | | |
| STOML2 sh#1 | CCGGGATGCAAGTCTTGATGAGGAACTCGAGTTCCTCATCAAGACTTGCATCTTTTTTG | AATTCAAAAAGATGCAAGTCTTGATGAGGAACTCGAGTTCCTCATCAAGACTTGCATC |
| STOML2 sh#2 | CCGGCCGTTATGAGATCAAGGATATCTCGAGATATCCTTGATCTCATAACGGTTTTTTG | AATTCAAAAACCGTTATGAGATCAAGGATATCTCGAGATATCCTTGATCTCATAACGG |
| **For ChIP assay** | | |
| HRE | CGATACTACGCTCTGGTG | AAGGCTGTGGTTGTTCTG |

Abbreviation: qPCR, quantitative real-time polymerase chain reaction; ChIP, Chromatin immunoprecipitation

| **Supplementary Table 3. Relationship between low and high STOML2 and Clinicopathologic features** | | | | | | | | | | | | | |  |
| --- | --- | --- | --- | --- | --- | --- | --- | --- | --- | --- | --- | --- | --- | --- |
| Variables | | | | STOML2 Level | | | | | | | |  | |  |
|  |  |  |  | High (*n* =105) | | | | Low (*n* =122) | | | |  | |  |
|  |  |  |  | No. of patients | | % | | No. of patients | | % | | ***P***  value | |  |
| **Age**, yrs^a^ | | | | 52.7 | | | | 51.9 | | | | 0.577 | |  |
| **Sex** | | | |  |  | | |  | |  | | 0.573 | |  |
| Male | | | | 93 | 88.6 | | | 105 | | 86.1 | |  | |  |
| Female | | | | 12 | 11.4 | | | 17 | | 13.9 | |  | |  |
| **Hepatitis B history** | | | | |  | | |  | |  | | 0.520 | |  |
| Yes | | | | 89 | 84.8 | | | 107 | | 87.7 | |  | |  |
| No | | | | 16 | 15.2 | | | 15 | | 12.3 | |  | |  |
| **HBeAg** | | | |  |  | | |  | |  | | 0.797 | |  |
| Positive | | | | 37 | 35.2 | | | 45 | | 36.9 | |  | |  |
| Negative | | | | 68 | 64.8 | | | 77 | | 63.1 | |  | |  |
| **AFP**,U/L^a^ | | | | 6793 | | | | 4450 | | | | 0.215 | |  |
| **ALT**, U/L^a^ | | | | 60.3 | | | | 49.8 | | | | 0.242 | |  |
| **Liver cirrhosis** | | |  | | | | |  | |  | | 0.865 | |  |
| Yes | | | | 85 | 81.0 | | | 100 | | 82.0 | |  | |  |
| No | | | | 20 | 19.0 | | | 22 | | 18.0 | |  | |  |
| **Microvascular invasion** | | | | |  |  | |  | |  | | 0.005 | |  |
| Yes | | | 54 | | 51.4 | | | 40 | | 32.8 | |  | |  |
| No | | | 51 | | 48.6 | | | 82 | | 67.2 | |  | |  |
| **Tumor size**^a^, cm | | | 5.9 | | | | | 5.3 | | | | 0.244 | |  |
| **Tumor number** | | | | | |  | |  | | | | 0.848 | |  |
| Single | | | 90 | | 86.7 | | | 106 | | 86.1 | |  | |  |
| Multiple | | | 15 | | 13.3 | | | 16 | | 13.9 | |  | |  |
| **Tumor encapsulation** | | | | | | | |  | |  | | 0.690 | |  |
| Complete | | | 50 | | 47.6 | | | 62 | | 50.8 | |  | |  |
| No | | | 55 | | 52.4 | | | 60 | | 49.1 | |  | |  |
| **Tumor differentiation** | | | | | |  |  | |  | | 0.312 | |  | |
| High | | 23 | | | 21.9 | | | 20 | | 16.4 | |  | |  |
| Low | | 82 | | | 78.1 | | | 102 | | 83.6 | |  | |  |
| **TNM stage** | | | | | | | |  | |  | | 0.070 | |  |
| Ⅰ | 49 | | | | 46.7 | | | 72 | | 59.0 | |  | |  |
| Ⅱ | 37 | | | | 35.2 | | | 39 | | 32.0 | |  | |  |
| Ⅲ | 19 | | | | 18.1 | | | 11 | | 9.0 | |  | |  |

^a^ Independent-samples *t*-test, Abbreviations: AFP: alpha fetoprotein; HBeAg: hepatitis B e antigen; TNM, Tumor-Node-Metastasis;
