# Supplementary figures and images for: STOML2 potentiates metastasis of hepatocellular carcinoma by promoting PINK1-mediated mitophagy and regulates sensitivity to lenvatinib
Source: J Hematol Oncol. 2021 Jan 14;14:16. doi: 10.1186/s13045-020-01029-3 (PMC7807703; doi:10.1186/s13045-020-01029-3)

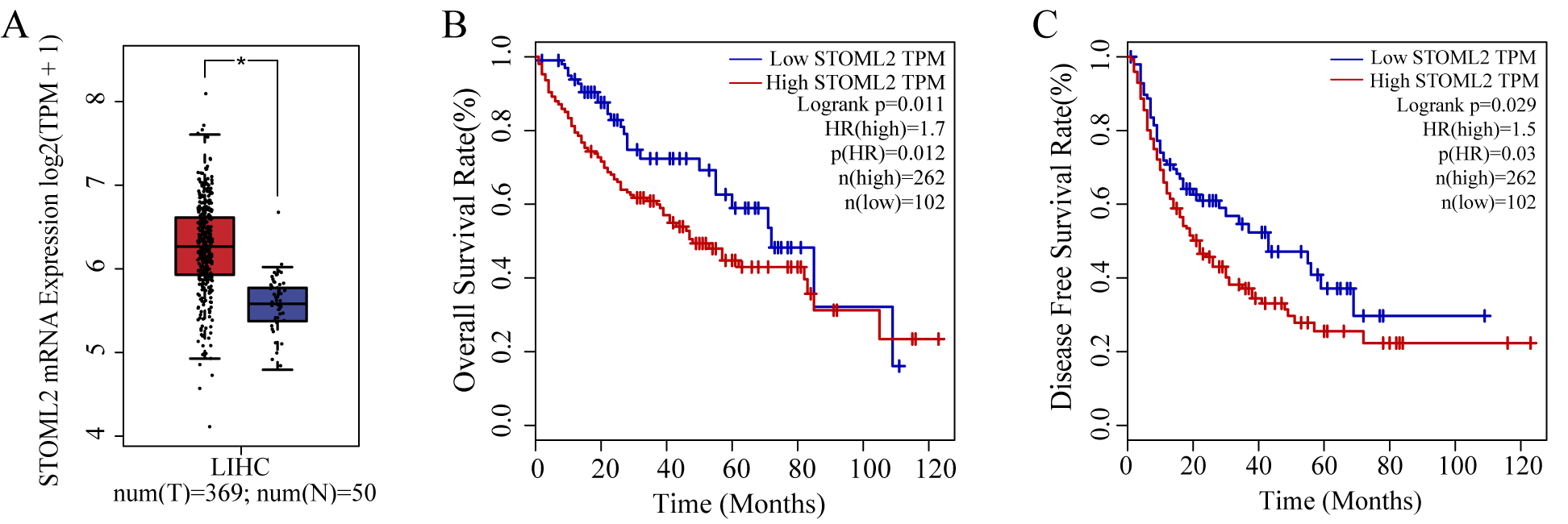

Supplement: Supplementary file 3 — Additional file 3: Figure S1. STOML2 expression is upregulated in HCC tissues and predicts a poor prognosis. (A) The mRNA expression level of STOML2 in HCC tissues (T, n=369) compared with the normal liver tissues (N, n=50) by the GEPIA database. (B-C) Patients with high STOML2 expression have poorer overall survival and disease free survival compared with patients with low STOML2 expression based on the RNA sequencing expression data (group cutoff in 28%/72%) from the TCGA project. *P<0.05. TPM transcripts per million, LIHC Liver hepatocellular carcinoma, HR hazard ratio. [file 13045_2020_1029_MOESM3_ESM.tif]

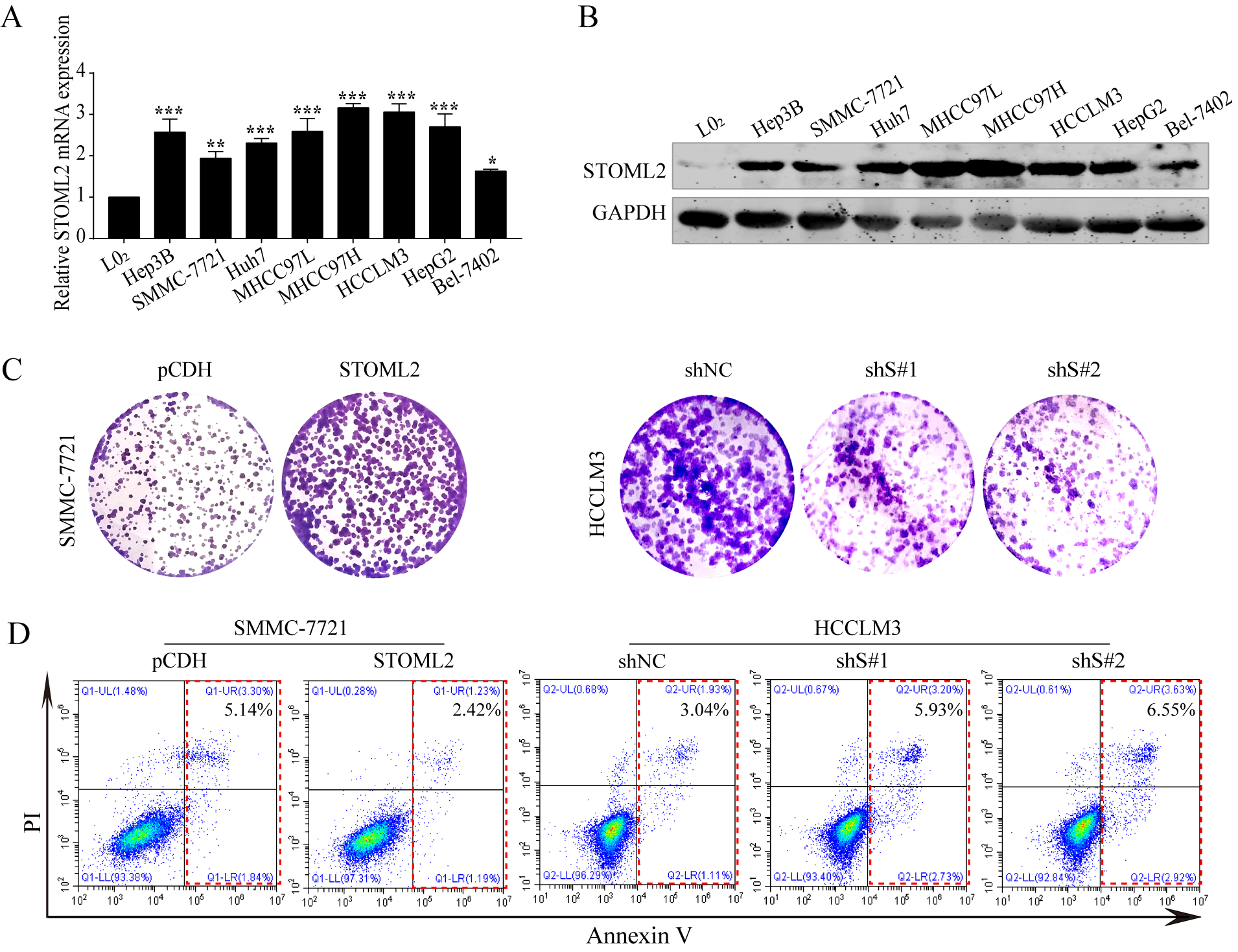

Supplement: Supplementary file 4 — Additional file 4: Figure S2. STOML2 expression is upregulated in HCC cells, promotes HCC proliferation and inhibits apoptosis in vitro. (A–B) Expression of mRNA (A) and proteins (B) levels of STOML2 in different HCC cell lines. Significantly increased STOML2 levels were detected in HCC cell lines especially in those with higher invasive and metastatic capabilities cells (MHCC-97H and HCCLM3) compared with L02. (C–D) The effects of STOML2 gain- or loss-of-function on in vitro proliferation (C) and apoptosis (D) by colony formation assay and flow cytometric analysis. *P<0.05; **P<0.01; ***P<0.001; ns, no significance. [file 13045_2020_1029_MOESM4_ESM.tif]

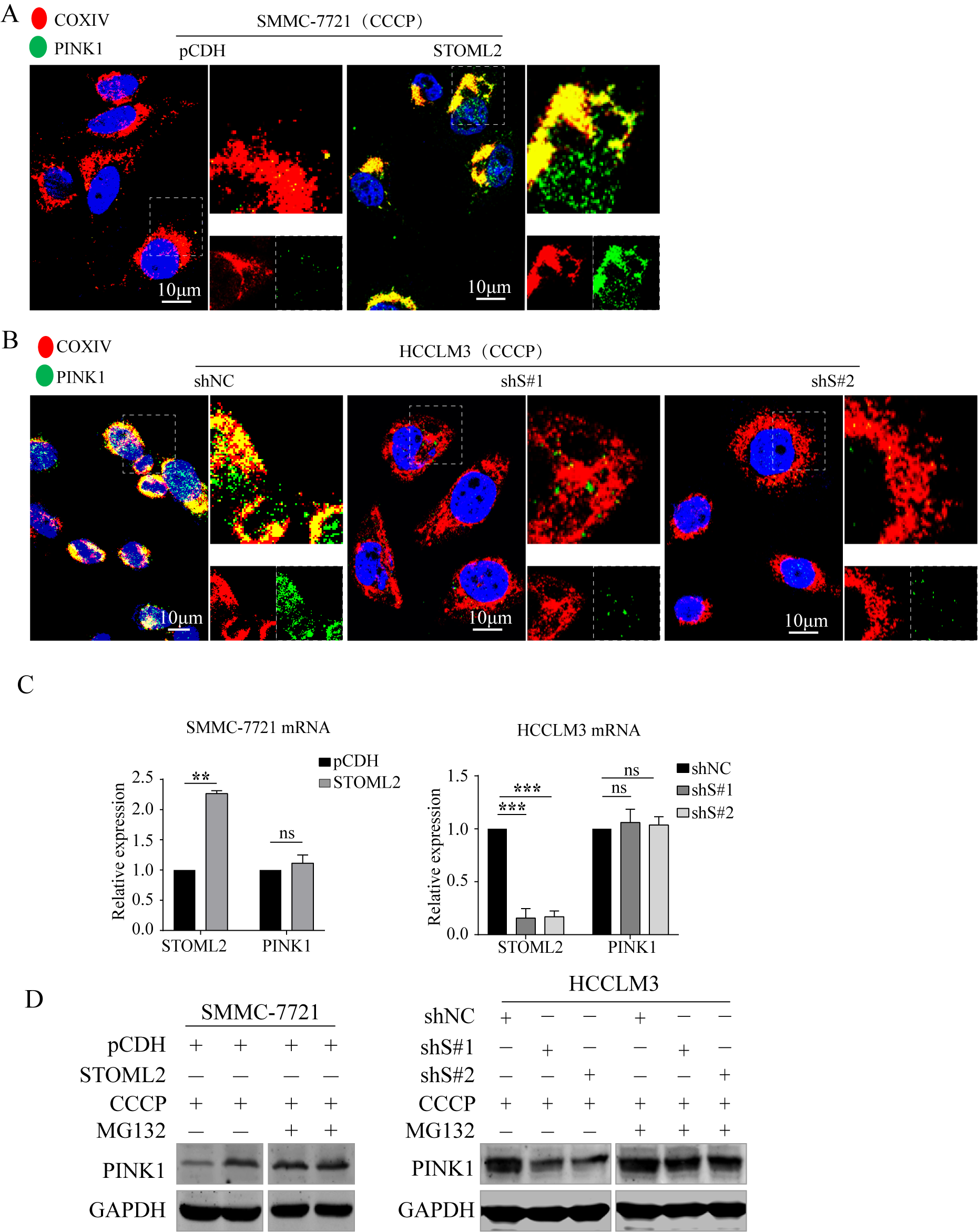

Supplement: Supplementary file 5 — Additional file 5: Figure S3. STOML2 promotes mitophagy in HCC cells under stress. (A–B) Confocal microscopy was performed to detect spatial colocalization of mitochondrial protein COXIV (red) and PINK1 (green) in SMMC-7721 and HCCLM3 control and derived cells under the treatment of CCCP (10 μM) for 4 h. (Scale bars:10 μm) (C) mRNA levels of STOML2 and PINK1 were determined by qRT-PCR in SMMC-7721 and HCCLM3 with manipulated the expression of STOML2, taking GAPDH mRNA as a control. (D) The alteration of PINK1 protein in SMMC-7721 and HCCLM3 control and derived cells with co-treatment of 10μM CCCP and 20 μM MG132 was detected by Western blot. **P<0.01; ***P<0.001; ns, no significance. [file 13045_2020_1029_MOESM5_ESM.tif]

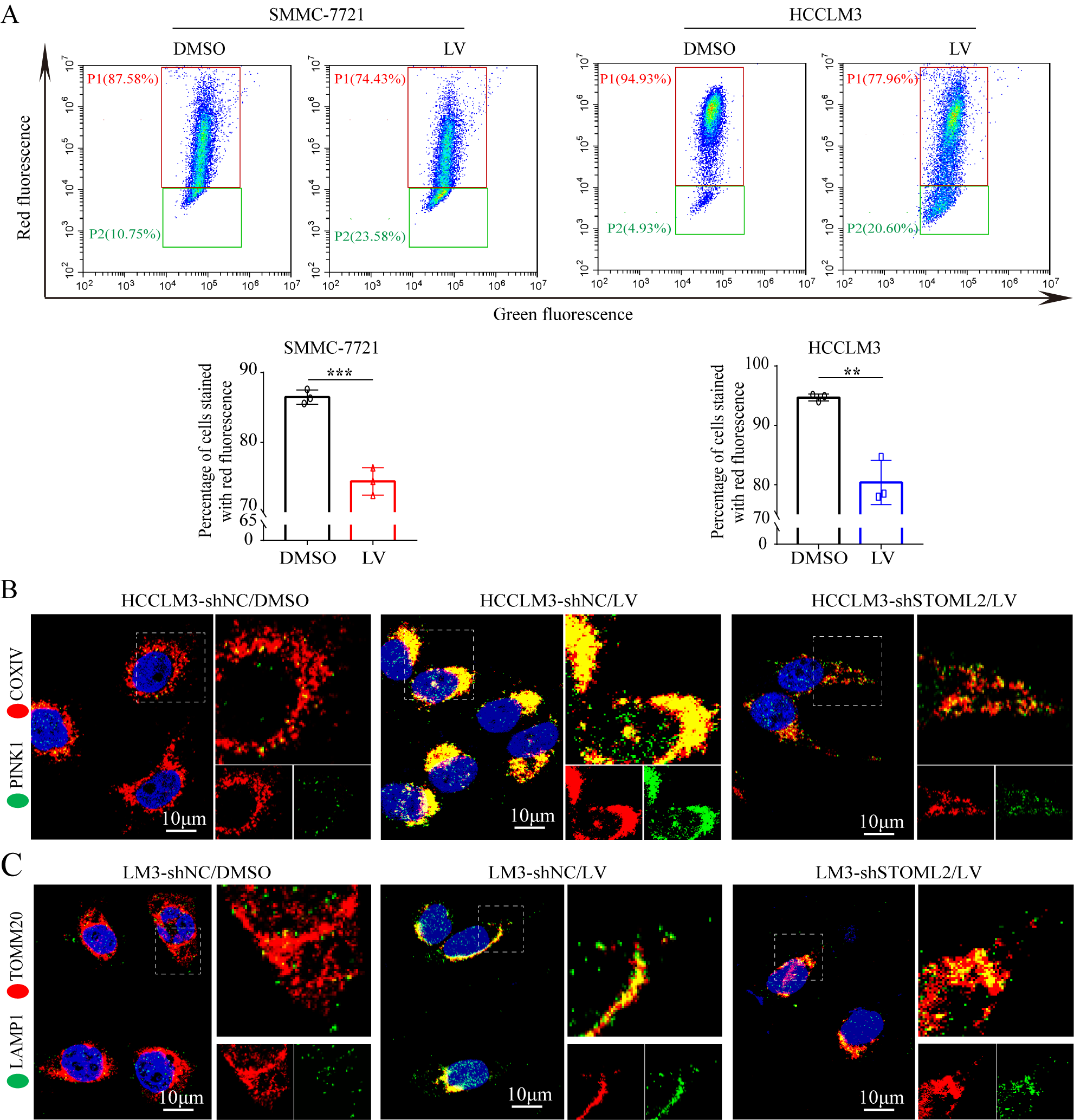

Supplement: Supplementary file 6 — Additional file 6: Figure S4. Lenvatinib induces cytoprotective mitophagy in HCC cells. (A) Double fluorescence staining of mitochondria by JC-1 was applied to monitor the mitochondrial membrane potential (MMP), as green fluorescent J-monomers indicating loss of MMP, and red fluorescent J-aggregates reflecting higher MMP. The MMP of SMMC-7721 and HCCLM3 with lenvatinib (10 μM, 4 h) treatment decreased significantly compared with controls by flow cytometric analysis. (B-C) Confocal microscopy was performed to detect spatial colocalization of mitochondrial protein COXIV (red) and PINK1 (green) (B) or TOMM20 (red) and LAMP1 (green) (Scale bars:10 μm) (C) in HCCLM3 cells with or without the treatment of lenvatinib (10 μM, 24 h). (Scale bars:10 μm) **P<0.01; ***P<0.001; ns, no significance. [file 13045_2020_1029_MOESM6_ESM.tif]

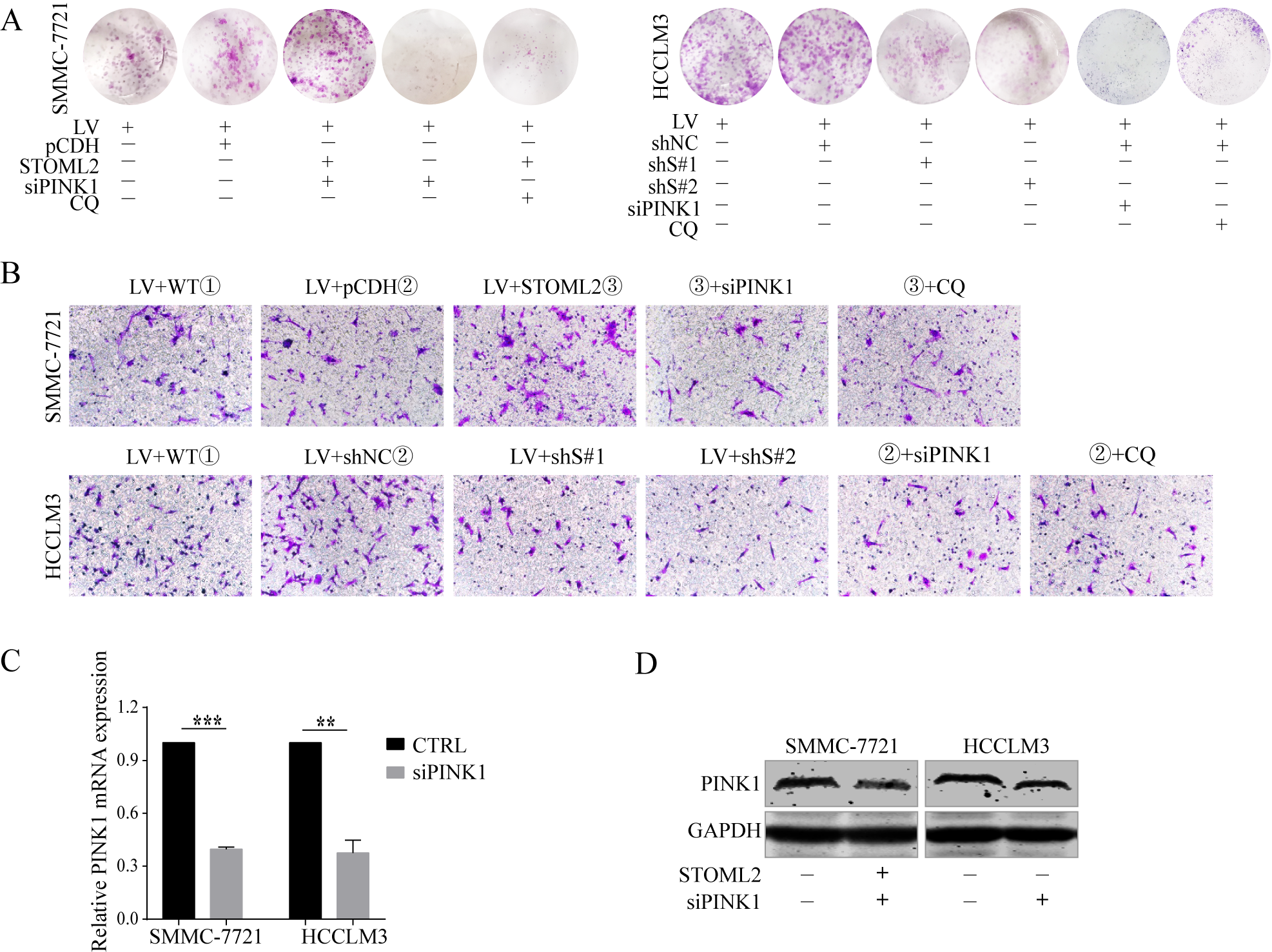

Supplement: Supplementary file 7 — Additional file 7: Figure S5. Inhibition of mitophagy suppresses HCC migration and sensitizes HCC cells to Lenvatinib treatment. (A–B) SMMC-7721 and HCCLM3 control and derived cells were co-treated with LV (5μM) and CQ (5μM) or transiently transfected with PINK1 siRNA. In STOML2-high expression cells, the effect of lenvatinib on inhibiting colony formation (A) and migration (B) of HCC cells was weakened while the inhibitory effect of lenvatinib was restored in when CQ treated or siPINK1 transfected cells. (C–D) Silencing PINK1 with short interfering RNA in SMMC-7721-STOML2 and HCCLM3. **P<0.01; ***P<0.001. [file 13045_2020_1029_MOESM7_ESM.tif]

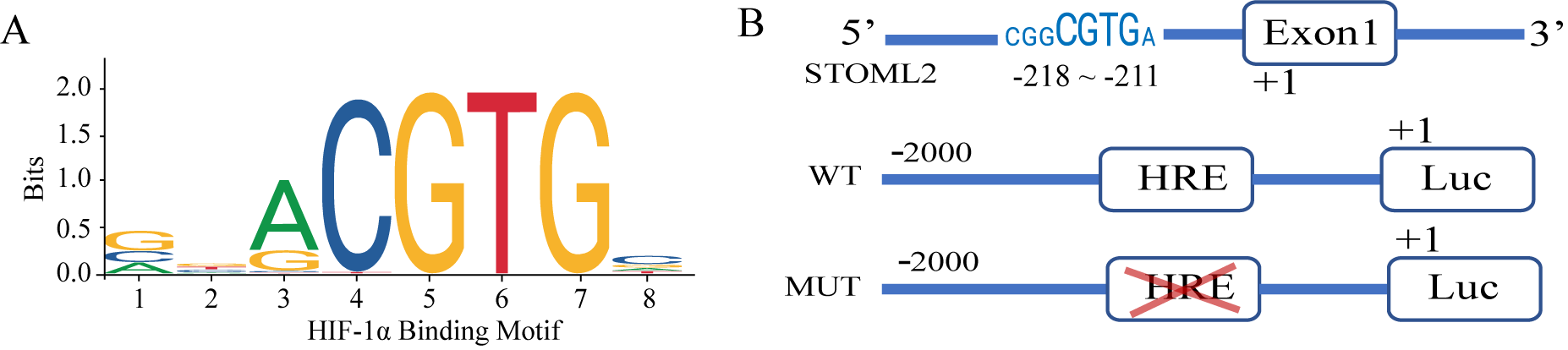

Supplement: Supplementary file 8 — Additional file 8: Figure S6. STOML2 is a target of HIF-1α. (A) HRE in the STOML2 promoter identified by the JASPAR database (http://jaspar.genereg.net/). (B) Schematic illustration of STOML2 promoter region with potential HIF-1α binding site. The WT and HRE mutant sequences were indicated. [file 13045_2020_1029_MOESM8_ESM.tif]
